# Serious Adverse Events Reporting in Phase III Randomized Clinical Trials of Colorectal Cancer Treatments: A Systematic Analysis

**DOI:** 10.3389/fphar.2021.754858

**Published:** 2021-11-18

**Authors:** Yanhong Yao, Zhentao Liu, Hua Zhang, Jian Li, Zhi Peng, Jinyu Yu, Baoshan Cao, Lin Shen

**Affiliations:** ^1^ Department of Medical Oncology and Radiation Sickness, Peking University Third Hospital, Beijing, China; ^2^ Department of Gastrointestinal Oncology, Peking University Cancer Hospital and Institute, Beijing, China; ^3^ Research Center of Clinical Epidemiology, Peking University Third Hospital, Beijing, China

**Keywords:** colorectal cancer, phase III clinical trial, reported proportion, real world, SAEs

## Abstract

**Objective:** The occurrence, development, and prognosis of serious adverse events (SAEs) associated with anticancer drugs in clinical trials have important guiding significance for real-world clinical applications. However, to date, there have been no studies investigating SAEs reporting in randomized clinical trials of colorectal cancer treatments. This article systematically reviewed the SAEs reporting of phase III randomized clinical trials of colorectal cancer treatments and analyzed the influencing factors.

**Methods:** We reviewed all articles about phase III randomized clinical trials of colorectal cancer treatments published in the PubMed, Embase, Medline, and New England Journal of Medicine databases from January 1, 1993, to December 31, 2018, and searched the registration information of clinical trials *via* the internet sites such as “clinicaltrials.gov”. We analyzed the correlation between the reported proportion (RP) of SAEs in the literature and nine elements, including the clinical trial sponsor and the publication time. Chi-square tests and binary logistic regression were used to identify the factors associated with improved SAEs reports. This study was registered on PROSPERO.

**Results:** Of 1560 articles identified, 160 were eligible, with an RP of SAEs of 25.5% (41/160). In forty-one publications reporting SAEs, only 14.6% (6/41) described the pattern of SAEs in detail. In clinical trials sponsored by pharmaceutical companies, the RP of SAEs was significantly higher than that in those sponsored by investigators (57.6 versus 20.7%, *p* < 0.001). From 1993 to 2018, the RP of SAEs gradually increased (none (0/6) before 2000, 17.1% (12/70) from 2000 to 2009, and 34.5% (29/84) after 2009). The average RP of SAEs published in the New England Journal of Medicine (N Engl J Med), the Lancet, the Journal of the American Medical Association (JAMA), the Lancet Oncology (Lancet Oncol), and the Journal of Clinical Oncology (J Clin Oncol) was significantly higher than that published in other journals (31.9 versus 16.7%, *p =* 0.030). In the clinical trials referenced by clinical guidelines, the RP of SAEs was higher than that in non-referenced clinical trials (32.0 versus 15.9%, *p =* 0.023). Binary logistic regression analysis showed that pharmaceutical company sponsorship, new drug research, and sample size greater than 1000 were positive influencing factors for SAEs reporting.

**Conclusion:** Although the RP of SAEs increased over time, SAEs reporting in clinical trials needs to be further improved. The performance, outcomes and prognosis of SAEs should be reported in detail to guide clinical practice in the real world.

## Introduction

Colorectal cancer is the third most commonly diagnosed malignancy worldwide (Fitzmaurice et al., 2017). Chemotherapy and targeted therapy play an important role in standard treatments for colorectal cancer. Fluorouracil-based adjuvant chemotherapy significantly improved the disease-free survival (DFS) and overall survival (OS) of stage II/III colorectal cancer (Group et al., 2007; André et al., 2009; Iveson et al., 2018). Combination chemotherapy with bevacizumab or cetuximab as the initial treatment significantly improved the median progression free survival (mPFS) and median overall survival (mOS) of metastatic colorectal cancer (Saltz et al., 2008; Van Cutsem et al., 2009; Qin et al., 2018). Fruquintinib and regorafenib in the 3 + line significantly prolonged the mOS and mPFS of advanced colorectal cancer (Grothey et al., 2013; Li et al., 2015; Li et al., 2018). Based on the results of clinical trials that have confirmed the efficacy of many chemotherapeutic and targeted drugs, experts have formed guidelines and consensuses to guide the diagnosis and treatment of colorectal cancer in the real world. Reporting the occurrence, development, and prognosis of adverse events (AEs), especially serious AEs (SAEs), is particularly crucial for reducing or avoiding the toxicity of regimens in real-world clinical practice, improving patients’ quality of life, and decreasing the psychological and economic burden of patients. During the past 20 years, SAEs have attracted increasing attention as the number of SAEs reported to the U.S. Food and Drug Administration (FDA) increased by 2.6 times from 1998 to 2005 (Moore et al., 2007) and by 2 times from 2006 to 2014 (Sonawane et al., 2018). Guidelines indicate that clinical trials should report AEs and SAEs in a consistent manner (Wallace et al., 2016).

AEs reporting is relatively higher in cancer clinical trial publications, but the reporting quality is low. A review showed that 96% of cancer clinical studies reported AEs, but oncology-specific reporting standards were lacking (Sivendran et al., 2014). Another article reviewed 325 randomized clinical trials, all of which reported the occurrence of AEs. Nevertheless, the AEs collection and analysis methods were highly heterogeneous, and the quality of AEs reporting did not improve significantly over time (Péron et al., 2013). In addition, there was a considerable discrepancy between the final published AEs data and the sponsors’ database (Scharf and Colevas, 2006). Although there have been some reviews of AEs reports, analysis of SAEs reports on colorectal cancer clinical trials is scarce, and the report proportion of SAEs in publications is unknown.

We systematically reviewed SAEs reporting from publications of colorectal cancer clinical trials, to further draw researchers’ attention to SAEs reporting. The SAEs reporting was influenced by many social factors, such as regional policy, preciseness and awareness of investigators, purpose of sponsor, so this article analyzed the possible influencing factors of SAEs reporting. Because the results of phase III randomized clinical trials were the most instructive in the real world, herein we just reviewed phase III randomized clinical trials.

## Materials and Methods

This study included randomized phase III colorectal cancer clinical trials whose intervention measures contained anticancer pharmaceuticals and whose results were published in PubMed, Embase, Medline, and the New England Journal of Medicine from January 1, 1993, to December 31, 2018. We analyzed several possible factors that may affect SAEs reporting in the literature. These factors included the region where the clinical trial was conducted, the sponsor of the clinical trial, whether the trial researched new drugs, the publication date which may reflect the change of policy and awareness of investigators, factors related with the rigorous of the clinical trials such as sample size, the type of journal and whether the clinical guidelines referenced the results of the study, and factors owned by clinical trials themselves, such as treatment line, therapeutic schedule.

### Literature Search Strategy

A review of citations from PubMed, Embase, Medline, and New England Journal of Medicine for studies published between January 1, 1993 and December 31, 2018, was performed to identify eligible colorectal cancer clinical trial publications for the analysis. The search terms were as follows: “colorectal cancer” [All fields] or “colon cancer” [All fields] or “rectal cancer” [All fields], and “phase 3” [All fields] or “phase III” [All fields]. We used the filters as follows: “subjects = cancer,” “article type = clinical trial,” “language = English,” “species = humans,” and “publications dates = 1/1/1993-12/31/2018.” Endnote X4 (Clarivate, Philadelphia, PA, United States) was used to manage the publications. We searched the registration information of clinical trials *via* the following internet sites: http://www.clinicaltrials.gov, http://www.isrctn.com/search, http://www.anzctr.org.au, https://www.umin.ac.jp/, http://apps.who.int/en/. The inclusion criteria were as follows: 1) phase III randomized colorectal cancer clinical trials, 2) intervention measure contained chemotherapy and/or target therapy, 3) the articles showed the efficacy and/or safety of the clinical trial, 4) published in English. The exclusion criteria were as follows: 1) the same research published repeatedly, 2) reviews, meta-analysis, molecular analysis and cost analysis, 3) subgroup analysis of the research already included, 4) intervention measure contained immune therapy (because the AEs spectrum of chemotherapy and immunotherapy is different), 5) clinical trials aimed to observe the efficacy or AEs of accompanying regimens along with anticancer therapy. The primary objective was the reported proportion (RP) of SAEs. The secondary objectives were the performance, outcomes and prognosis of SAEs.

$$\mathrm{RP of SAEs=}\frac{\mathrm{Publications that reported SAEs}}{\mathrm{All eligible publications}}\mathrm{\times 100\%}$$

### The Criteria of AEs, SAEs and SAE Reporting

According to the National Cancer Institute Common Toxicity Criteria (NCI-CTC) version 5.0 (Common terminology criteria for adverse events (CTCAE) v5.0, 2017), an AE is any unfavorable and unintended sign (including an abnormal laboratory finding), symptom, or disease temporally associated with the use of a medical treatment or procedure that may or may not be considered related to the medical treatment or procedure. Grade 3 AEs are defined as: 1) severe or medically significant but not immediately life-threatening, 2) hospitalization or prolongation of hospitalization indicated, 3) disabling, 4) limiting self-care activities of daily living (ADL). Grade 4 AEs are defined as: 1) life-threatening consequences, 2) urgent intervention indicated. Grade 5 AEs are death related to AEs.

SAEs were diagnosed according to NCI-CTC version 5.0 (Common terminology criteria for adverse events (CTCAE) v5.0, 2017) as follows:

An SAE is any untoward medical occurrence that, at any dose: 1) results in death, 2) is life-threatening, 3) an event is considered life-threatening if it is suspected that the individual is at substantial risk of dying at the time of the AEs, 4) requires inpatient hospitalization or prolongation of existing hospitalization (an admission and/or overnight stay or an event that prolongs hospitalization), 5) results in persistent or significant disability/incapacity (includes an AEs that resulted in a substantial disruption of a person’s ability to conduct normal life functions, i.e., significant, persistent or permanent change in, impairment of, damage to or disruption in the individual’s body function/structure, physical activities, and/or quality of life), 6) is a congenital anomaly/congenital disability, 7) is medically significant (other important medical events may be considered serious when, based on appropriate medical judgment, they might jeopardize the individual and/or may require medical or surgical intervention to prevent the event from meeting a criterion for an SAE).

Herein we mainly discussed the SAEs reporting. If the publication pointed out the occurrence of SAEs, even the incidence was zero, it was judged to have reported SAEs. SAEs consisted of many events not only death, so if the publication just only reported death and didn’t mention “SAEs,” it wasn’t judged to have reported SAEs in this review. And reporting Grade 3/4 AEs were not identified as having reported SAEs.

### Data Extraction

The data were collected independently by two investigators (Yanhong Yao and Zhentao Liu) who screened eligible publications and searched the registry of clinical trials. The collected data included performance, outcomes and prognosis of SAEs, the region where the clinical trial was conducted, the sponsor of the clinical trial, whether the trial researched new drugs, the sample size, the publication date, the type of journal, whether the clinical guidelines [including the National Comprehensive Cancer Network (NCCN), American Society of Clinical Oncology (ASCO), European Society for Medical Oncology (ESMO) and Chinese Society of Clinical Oncology (CSCO)] referenced the study results, the treatment lines, and treatment schedules. Professor Baoshan Cao checked the data if inconsistencies existed between the results collected by the two investigators.

### Statistical Analysis

We used SPSS version 19.0 (IBM, New York, United States) to analyze the data, and differences were considered statistically significant when the two-sided *p* values were less than 0.05. Frequencies and percentages were calculated for counting data. The chi-square test was used to assess the association between RPs of SAEs and collected items, and Fisher’s exact test was used if the theoretical number was less than 5 or the sample size was less than 40. A binary logistic regression model was used to identify items associated with SAEs reporting. The dependent variable was whether reported SAEs, and the independent variables were the positive influencing factors for SAEs reporting based chi-square test. The method of the independent variables entering the regression equation was “Backwald”.

## Results

### Characteristics of Selected Publications

**FIGURE 1:**
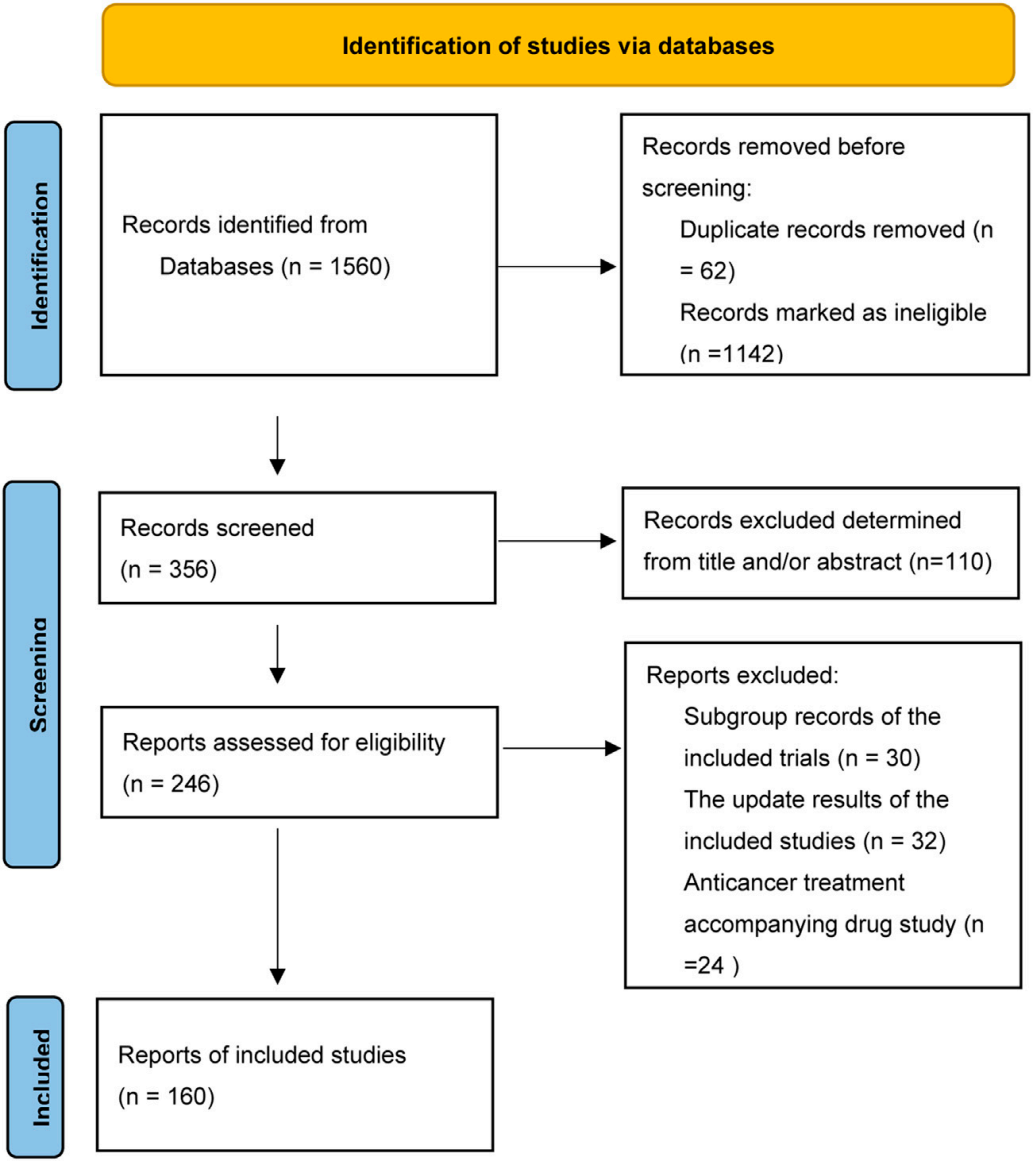
The flowchart of the studies included in this analysis.

From the 1560 publications initially collected by the two investigators, a total of 160 publications (Supplementary Material) were included in this analysis according to the eligible criteria (Figure 1). The characteristics of the 160 included publications were listed in Table 1. There were more trials conducted in local region (139, 86.9%) than worldwide (21, 13.1%). Ninety-four (58.8%) articles were published in journals such as N Engl J Med, Lancet, JAMA, Lancet Oncol and J Clin Oncol, and one hundred fifty-four (96.3%) articles were published after 2000. The sample size of one hundred and twenty (75.0%) articles was greater than 300. One hundred and six (66.2%) clinical trials researched treatment of metastatic colorectal cancer.

**TABLE 1:** Characteristics of enrolled articles.

Characteristic	n	%
Region of clinical trials conducted		
Worldwide	21	13.1
Local region	139	86.9
Year of publication		
Before 2000	6	3.8
2000-2009	70	43.8
After 2009	84	52.5
Journals		
N Engl J Med	8	5.0
Lancet	9	5.6
JAMA	2	1.3
Lancet Oncol	19	11.9
J Clin Oncol	56	35.0
Ann Oncol	29	18.1
Eur J Cancer	9	5.6
Br J Cancer	7	4.4
Others	21	13.1
Sample size		
<300	40	25.0
300-999	79	49.4
≥1000	41	25.6
Treatment line		
Adjuvant/Neoadjuvant	54	33.8
First-line	81	50.6
Second-line and above	25	15.6
Total	160	

### The Performance, Outcomes and Prognosis of SAEs

Forty-one (25.5%) of the 160 included publications reported SAEs (Table 2). Only six publications described the performance of SAEs in detail. None described the detailed treatment process for the SAEs. All of the publications that reported SAEs listed grade 3/4 AEs (Table 3). Grade 3/4 hematological toxicity (40/41) and gastrointestinal reactions (37/41) were the most common. Hypertension, proteinuria, and gastrointestinal perforation were more common for anti-vascular drugs. Hand-foot syndrome (HFS) was more common for capecitabine and regorafenib. Skin reactions were more common for cetuximab and panitumumab.

**TABLE 2:** The articles reported SAEs.

Clinical Trial Register No.	Authors (Year of publication)	Comparative Regimens	Sample size	Reported proportion of SAE
NCT00335595	Eduardo et al. (2012)	XELOX + Bev/Bev	480	14%/20%
NCT00719797	Loupakis et al. (2014)	FOLFIRI + Bev/FOLFOXIRI + Bev	508	19.7%/20.4%
NCT00154102	Van Cutsem et al. (2009)	FOLFIRI + Cet/FOLFIRI	1198	26%/19.3%
NCT00749450/ISRCTN59757862	Iveson et al. (2018)	Oxaliplatin-Fluoropyrimidine 3 month/6 month	6088	14%/16%
NCT00724503/NCT01721954	Wasan et al. (2017)	FOLFOX/FOLFOX + SIRT	1102	43%/54%
ISRCTN45133151	Kerr et al. (2016)	Capecitabine + Bev/Capecitabine	1941	30%/20%
NCT01584830	Li et al. (2015)	Regorafenib + BSC/Placebo + BSC	204	32%/26%
NCT00700102	Bennouna et al. (2013)	Bev + Chemotherapy/Chemotherapy	409	32%/33%
NCT00484939	Cunningham et al. (2013)	Bev + Capecitabine/Capecitabine	280	30%/31%
NCT01996306	Xu et al. (2018)	XELIRI ± Bev/FOLFIRI ± Bev	650	15%/20%
NCT00112918	De Gramont et al. (2012)	FOLFOX or XELOX + Bev/FOLFOX	2867	26%, 25%/20%
NCT01103323	Grothey et al. (2013)	Regorafenib/Placebo	760	44%/40%
NCT00005586/ISRCTN82375386	QUASAR Collaborative Group et al. (2007)	Fu/Observe	3,239	0.5%/0.25%
NCT02314819	Li et al. (2018)	Fruquintinib/Placebo	404	15.5%/5.8%
NCT01955837	Xu et al. (2018)	Trifluridine or Tipiracil (TAS-102)/Placebo	406	23.2%/23.0%
NCT01228734	Qin et al (2018)	Cet + FOLFOX4/FOLFOX4	553	19.1%/13.1%
NCT00724503	Van Hazel et al. (2016)	mFOLFOX6 ± Bev/mFOLFOX6 ± Bev + Radiation	530	41.6%/54.1%
NCT00384176	Schmoll et al. (2012)	FOLFOX + Cediranib/FOLFOX + Bev	1422	39%/33%
NCT00399035	Hoff et al. (2012)	Cediranib + FOLFOX or CAPOX/Placebo + FOLFOX or CAPOX	1076	40.8%/29.3%
NCT00056459	Hecht et al. (2011)	PTK787/ZK 222584 + FOLFOX4/Placebo + FOLFOX4	1168	46.8%/38.2%
NCT00056446	Van Cutsem et al. (2011)	FOLFOX4+ PTK787/ZK 222584/FOLFOX4+Placebo	855	45.0%/34.5%
NCT00339183	Peeters et al. (2010)	FOLFIRI + Pan/FOLFIRI	1186	WT41%/31%
				MT37%/30%
NCT00364013	Douillard et al. (2010)	FOLFOX/FOLFOX + Pan	1096	WT36%/40%
				MT29%/47%
NCT00063141	Sobrero et al. (2008)	CPT11/CPT11 + Cet	1298	22.6%/29.2%
NCT00069121	Schmoll et al. (2007)	XELOX/FOLFOX	1886	22.1%/24.6%
NA	Porschen et al. (2007)	CAPOX/FOLFOX	476	21%/24%
NCT00004885	Kohne et al. (2005)	IRI + FuFA/FuFA	430	8%/3%
NA	Tournigand et al. (2004)	FOLFIRI Followed by FOLFOX6 or the Reverse Sequence	220	First line14%/5%
				Second line 6%/4%
NA	Saini et al. (2003)	LV5Fu2/mFULU	905	4.6%/5.1%
NCT00115765	Hecht et al. (2009)	FOLFOX or FOLFIRI + Bev/LFOX or FOLFIRI + Bev + Pan	1053	Panitumumab-related 19%
NCT01661270	Li et al. (2018)	Aflibercept + FOLFIRI/Mixed strategy/Placebo + FOLFIRI	332	20%/13%/15%
NCT01030042	Cascinu et al. (2017)	IRI + Cet Folllowed by FOLFOX or the Reverse	110	18%/10%
NA	Köhne et al. 2013	5-Fu/FA + high dose Fu	1601	14.5%/15.8%
ISRCTN2194324	Popova et al. (2008)	FuLV/Raltitrexed	1921	18.3%/16.3%
NCT00642577	Guan et al. (2011)	mIFL/mIFL + Bev	214	18.6%/10%
ACTRN12610 000148077	Papadimitriou et al. (2011)	FOLFIRI/LV5Fu2	873	27%/18%
NCT02149108	Van Cutsem et al. (2018)	Nintedanib/Placebo	768	39%/35%
NCT00646607	Lonardi et al. (2016)	FOLFOX4/XELOX	3,759	4.2%/5.6%
NCT00720512	Masi et al. (2015)	FOLFIRI/FOLFOX + Bev	185	7%/7%
NA	Fields et al. (2009)	Fu/Fu + Edrecoloma	1839	26%/26%
NCT00143403	Ychou et al. (2009)	FuLV/FOLFIRI	153	6%/13%

Abbreviation: NA, not available; Bev, bevacizumab; Pan, panitumumab; Cet, cetuximab; Fu, fluorouracil; CPT11, irinotecan; LV, leucovorin; FA, folinic acid; XELOX, oxaliplatin + capecitabine; CAPOX, oxaliplatin + capecitabine; FOLFOX, bolus and infusional fluorouracil/leucovorin + oxaliplatin; FOLFIRI, bolus and infusional fluorouracil/leucovorin; FOLFOXIRI, bolus and infusional fluorouracil/leucovorin + oxaliplatin + irinotecan; XELIRI, irinotecan + capecitabine; IFL, fluorouracil + leucovorin + irinotecan; SIRT, selective internal radiotherapy; BSC, best supportive care; PTK787 ZK: an Oral Vascular Endothelial Growth Factor Receptor Inhibitor; WT, wide-type; MT, mutant.

**TABLE 3:** Details of Grade 3/4 AEs in the articles reported SAEs.

Comparative Regimens/AEs	Haema-AEs	FN	Infection	GIR	Liver injury	GIP	TEE	Hyper-tension	Cardio-toxicity	Haem-orrhage	Hema-turesis	Protein-uria	WHC	SNP	HFS	Asthenia	Cutire-action	Anap-hylaxis	Dysp-noea
Regorafenib/Placebo	+			+	+			+				+		+	+		+		+
Bev + Chemotherapy/Chemotherapy	+		+	+		+	+	+		+				+		+			+
FOLFOX-4/XELOX	+	+		+										+		+		+	
FOLFIRI + Cet/FOLFIRI	+			+													+		
FOLFOX/IRI + Cet	+		+	+	+									+		+	+		
CPT11/CPT11+Cet	+			+												+	+		
FU/FU + Edrecoloma	+			+												+	+		
3 versus 6 months of adjuvant oxa-fluoropyrimidine	+													+	+		+		
Regorafenib + BSC/Placebo + BSC	+		+		+			+	+	+		+			+		+		
FOLFOX + Pan/FOLFOX	+	+		+			+							+			+		
FOLFIRI followed by FOLFOX6 or the Reverse Sequence	+	+		+										+			+		
FOLFOX, FOLFIRI + Bev/FOLFOX or FOLFIRI + Bev + Pan	+		+	+			+	+									+		
FOLFIRI + Pan/FOLFIRI	+	+		+			+										+		
Cet + FOLFOX-4/FOLFOX-4	+			+													+		
FOLFIRI + Bev/FOLFOXIRI + Bev	+	+		+			+	+						+		+			
FOLFOX4+PTK/ZK/FOLFOX4+Placebo	+			+				+				+				+			
Bev + Capecitabine/Capecitabine	+			+			+	+	+	+						+			
Trifluridine/Tipiracil(TAS-+02)/placebo	+		+	+	+			+								+			
FU/LV/FOLFIRI	+			+												+			
FOLFOX or XELOX + Bev/FOLFOX	+					+	+	+		+			+	+	+				
XELOX + Bev/Bev	+			+		+	+	+	+	+		+		+	+				
Cediranib + FOLFOX/CAPOX/Placebo + FOLFOX/CAPOX				+				+						+	+				
LV5FU2/mFU/LV	+	+	+	+			+							+	+				
XELOX/FOLFOX	+	+		+										+	+				
Capecitabine + Bev/Capecitabine	+			+		+	+	+		+		+	+		+				
XELIRI ± Bev/FOLFIRI ± Bev	+	+	+	+		+	+	+		+		+			+				
Fruquintinib vs Placebo	+		+	+	+			+		+		+			+				
FUFA/FOLFIRI	+	+		+				+							+				
FOLFOX + CEDIRANIB/FOLFOX + Bev	+			+		+	+	+		+		+		+					
Nintedanib/Placebo	+		+	+	+			+		+		+		+					
FOLFOX/FOLFOX + SIRT	+	+		+	+		+			+				+					
mFOLFOX6 ± Bev/mFOLFOX6 ± Selective Internal Radiation	+	+		+	+		+							+					
FOLFIRI/LV5FU2	+	+		+	+									+					
PTK787/ZK 222584 +FOLFOX4/Placebo + FOLFOX4	+			+			+	+						+					
mIFL/mIFL + Bev	+	+		+		+	+	+		+		+							
Aflibercept + FOLFIRI/Placebo + FOLFIRI	+			+				+		+		+							
FOLFIRI/FOLFOX + Bev	+			+		+	+	+		+	+								
Fu/Observe	+			+															
CAPOX/FUFOX	+																		
FU/LV/Tomudex	+	+		+															
Bolus 5-FU/FA HD-FU	+			+											+				

Abbreviation: AEs, adverse events; SAE, serious adverse events; Bev, bevacizumab; Pan, panitumumab; Cet, cetuximab; Fu, fluorouracil; CPT11, irinotecan; LV, leucovorin; FA, folinic acid; XELOX, oxaliplatin + capecitabine; CAPOX, oxaliplatin + capecitabine; FOLFOX, bolus and infusional fluorouracil/leucovorin + oxaliplatin; FOLFIRI, bolus and infusional fluorouracil/leucovorin; FOLFOXIRI, bolus and infusional fluorouracil/leucovorin + oxaliplatin + irinotecan; XELIRI, irinotecan + capecitabine; IFL, fluorouracil + leucovorin + irinotecan; SIRT, selective internal radiotherapy; BSC, best supportive care; PTK787 ZK: an oral vascular endothelial growth factor receptor inhibitor; Haema-AEs, haematological adverse events; FN, febrile neutropenia; GIR, gastrointestinal reaction; GIP, gastrointestinal perforation; TEE, thromboembolic events; WHC, wound-healing complications; SNP, sensory neuropathy; HFS, hand food syndrome.

**FIGURE 2:**
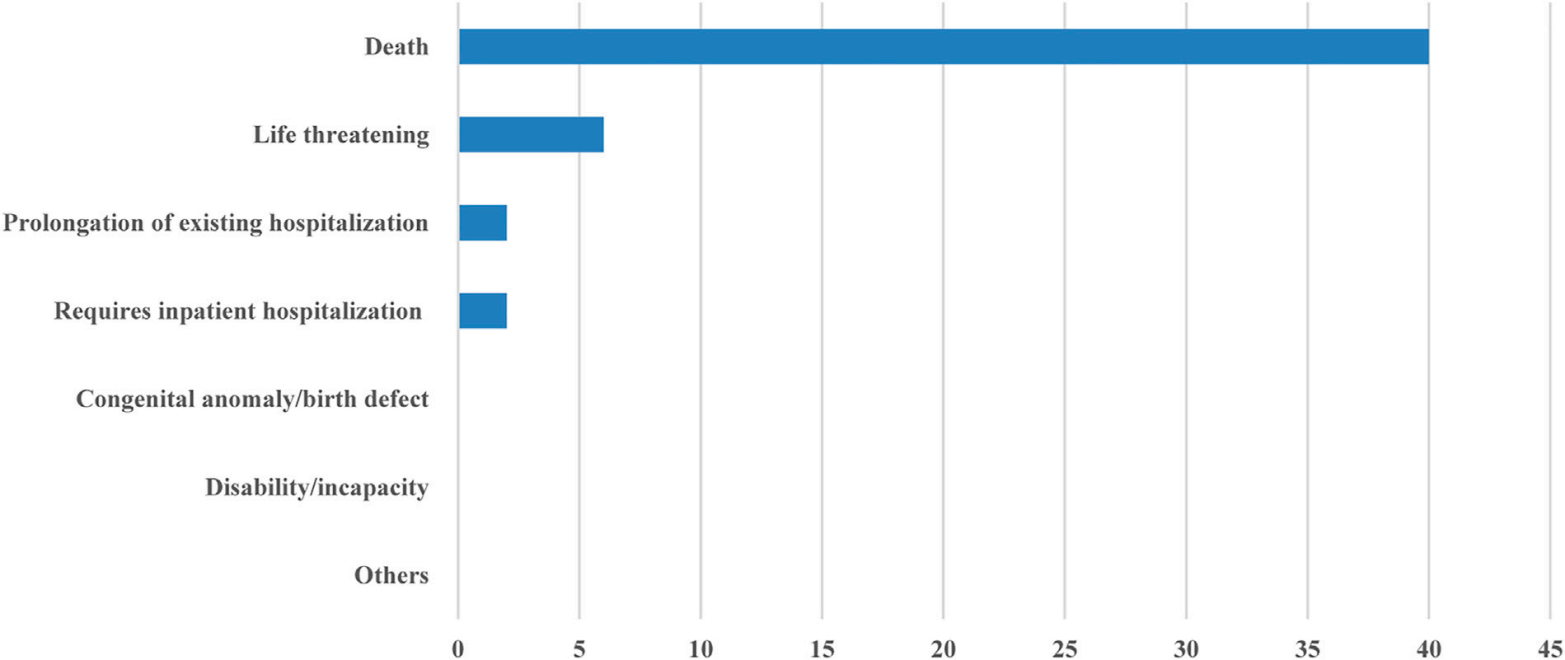
Number of publications reported the outcomes of SAEs.

Of the forty-one publications that reported SAEs, forty publications reported whether the SAEs resulted in death, and thirty-seven publications reported the relationship between death and the treatment, and only fifteen reported the relationship between the non-death SAEs and the anticancer treatment. The proportion of deaths caused by SAEs was as follows: less than 1% in nineteen clinical trials, 1–5% in sixteen clinical trials, and 5–10% in five clinical trials. Six publications reported whether the SAEs were life-threatening, and only two publications reported the prognosis of SAEs in detail (Figure 2).

### Analysis of the RP of SAEs

**FIGURE 3:**
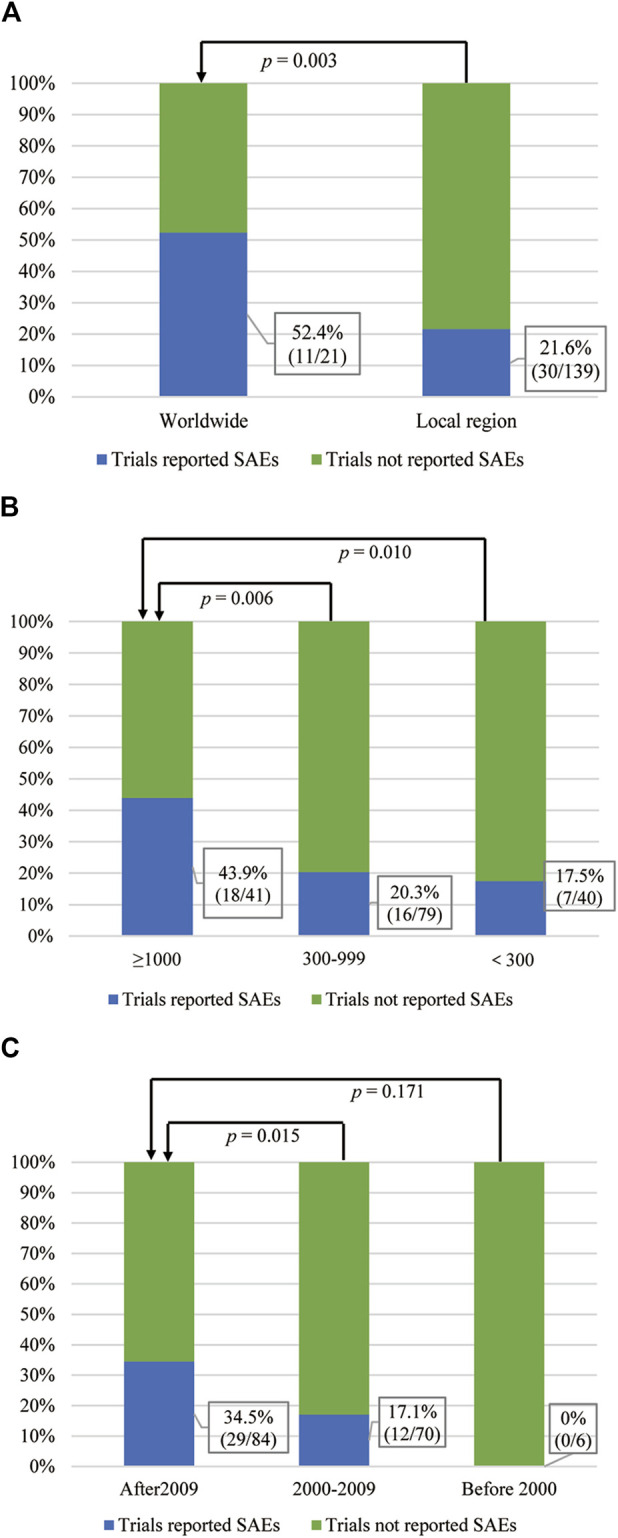
The correlation between the RP of SAEs and the influencing factors. **(A)** Region where clinical trials conducted and SAEs reported status. **(B)** Sample size and SAE reported status. **(C)** Publication time and SAEs reported status.

**FIGURE 4:**
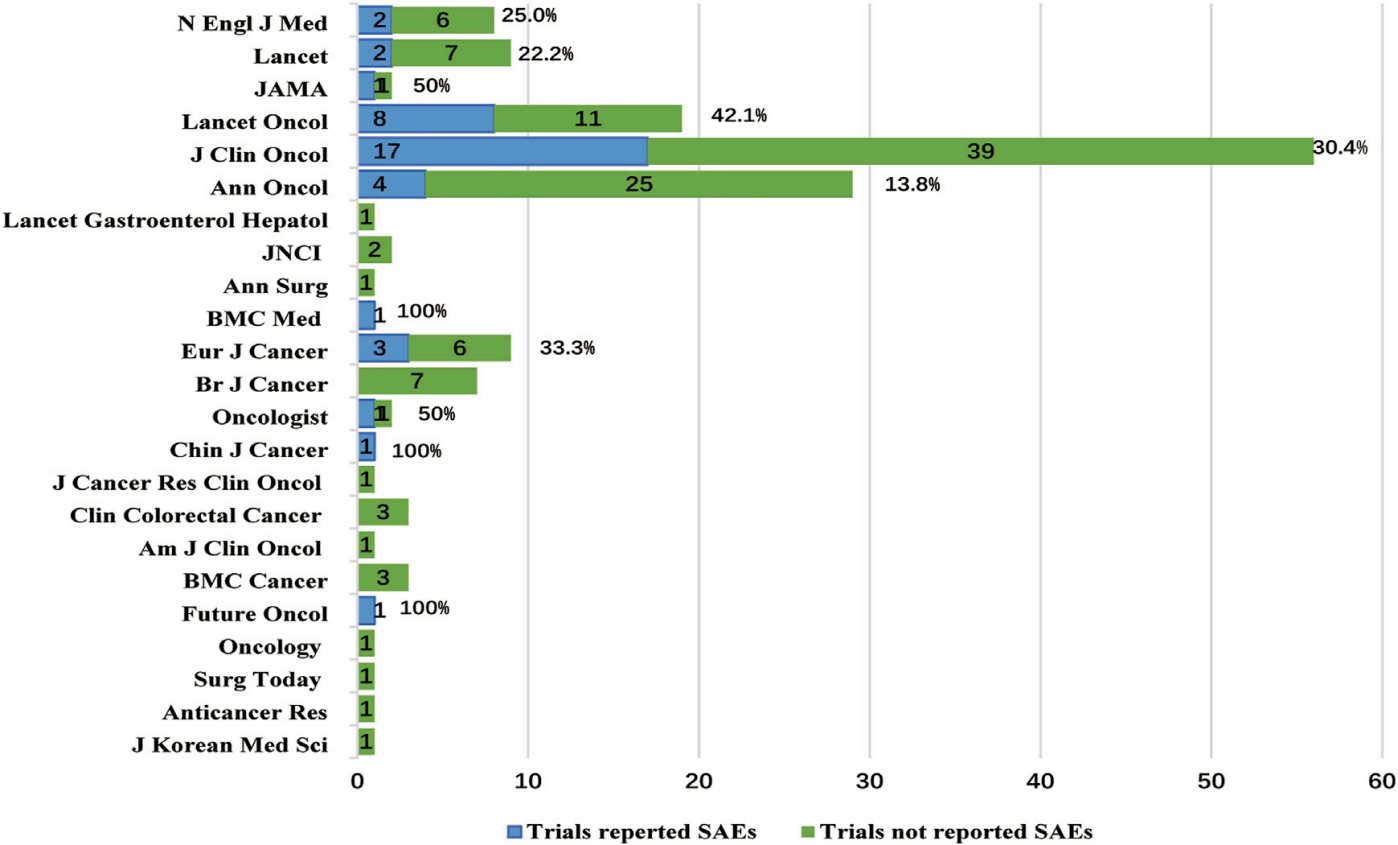
Journals and SAEs reported status.

Chi-square tests (Table 4) showed that the RP of SAEs in clinical trials conducted worldwide (52.4% [11/21]) was higher than conducted in local region (21.6% [30/139], *p =* 0.003, Figure 3A). The RP of SAEs was more than twice in clinical trials sponsored by pharmaceutical companies (57.6% [19/33]) as much as sponsored by investigators (20.7% [17/82], *p <* 0.001). Clinical trials examining new drugs (45.5% [25/55]) liked to report SAEs more than those not examining new drugs (15.2% [16/105], *p <* 0.001). Clinical trials with larger sample sizes (≥1000, 43.9% [18/41]) seemed to have a greater RP of SAEs than those with medium sample sizes (300–999, 20.3% [16/79], *p =*0.006) and small sample sizes (*<*300, 17.5% [7/40], *p =* 0.010, Figure 3B). The RP of SAEs increased over time. The RP of SAEs in articles published after 2009 (34.5% [29/84]) was higher than that published from 2000 to 2009 (17.1% [12/70], *p =* 0.015) and published before 2000 (none [0/6], *p =* 0.171, Figure 3C). The RP of SAEs in clinical trials whose results were referenced by the guidelines (32.0% [31/97]) was greater than that not referenced by guidelines (15.9% [10/63]) (*p =* 0.023). The RPs of SAEs in studies published in famous journals were as follows: 25% [2/8] in N Engl J Med, 22.2% [2/9] in Lancet, 42.1% [8/19] in Lancet Oncol, 50.0% [1/2] in JAMA and 30.4% [17/56] in J Clin Oncol (Figure 4), with an average RP of SAEs of 31.9% (30/94), which was significantly higher than that in studies published in other journals (16.7%, [11/66], *p =* 0.030). The RP of SAEs was significantly higher in clinical trials about second line and above treatment than those about first line and adjuvant/neoadjuvant treatment, and higher in clinical trials researched targeted therapy ± chemotherapy than those researched other therapeutic schedules (Table 4).

**TABLE 4:** Analysis of the influencing factors of SAEs reporting.

Characteristic	Trials	Trials reported SAEs	RP of SAEs (%)	*p* Value
Total	160	41	25.5	
Region of clinical trials				
Worldwide	21	11	52.4	Reference
Local region	139	30	21.6	0.003
Trial sponsor				
Pharmaceutical Company	33	19	57.6	Reference
Investigator	82	17	20.7	<0.001
Unknown	45	5	11.1	<0.001
New drug study				
Yes	55	25	45.5	Reference
No	105	16	15.2	<0.001
Sample size				
≥1000	41	18	43.9	Reference
300-999	79	16	20.3	0.006
<300	40	7	17.5	0.010
Year of publication				
After 2009	84	29	34.5	Reference
2000-2009	70	12	17.1	0.015
Before 2000	6	0	0	0.171
N Engl J Med, Lancet, Lancet Oncol, JAMA, J Clin Oncol				
Yes	94	30	31.9	Reference
No	66	11	16.7	0.030
Referenced by Guidelines				
Yes	97	31	32.0	Reference
No	63	10	15.9	0.023
Treatment line				
Second-line and 2nd +	25	11	44.0	Reference
First-line	81	18	22.2	0.033
Adjuvant/Neoadjuvant	54	12	22.2	0.048
Therapeutic schedule				
Targeted therapy ± Chemotherapy	63	23	36.5	Reference
Chemotherapy ± Others	97	18	16.7	0.011

Abbreviation: RP, Report Proportion; SAEs, Serious Adverse Events.

After adjusting for the nine factors, logistic regression analysis showed that pharmaceutical company sponsorship, new drug research and a sample size greater than 1000 were positive influencing factors for SAEs reporting (Table 5).

**TABLE 5:** Binary logistic regression analysis of influencing factors of SAEs reporting.

Characteristic	Regression coefficient β	Standard error	Wald	Sig	Exp(B)	Exp(B) (95%CI)
Trial sponsor						
Pharmaceutical Company	Reference					
Investigator	−1.304	0.481	7.352	0.007	0.271	0.106–0.697
Unknown	−1.478	0.670	4.859	0.028	0.228	0.061–0.849
New drug study						
Yes *vs* No	−1.128	0.450	6.284	0.012	0.324	0.134–0.782
Sample size						
≥1000	Reference					
300-999	−1.059	0.476	4.955	0.026	0.347	0.136–0.881
<300	−1.120	0.580	3.730	0.053	0.326	0.105–1.017
Year of publications						
After 2009	Reference					
2000-2009	−0.833	0.506	2.714	0.099	0.435	0.161–1.171
Before 2000	−19.719	16,407	0.0	0.999	0	0
Referenced by Guidelines						
Yes *vs* No	2.063	0.887	5.415	0.020	7.872	0.181–1.900
Therapeutic schedule						
Targeted therapy ± Chemotherapy *vs* Chemotherapy ± Others	1.241	0.870	2.034	0.154	3.458	0.628–19.029

Abbreviation: SAEs, serious adverse events.

## Discussion

The registration rate of oncology clinical trials has significantly increased since 2005 (Song and Kim, 2020), and the number of clinical trials for anticancer drugs has also increased in the past decade in China (Li et al., 2019). The China Food and Drug Administration (CFDA) has issued a series of innovations to accelerate new agent approvals in oncology (Wang, 2017). Randomized phase III clinical trials are considered to be the gold standard in clinical practice. Therefore, clinical trials and SAEs reports lay the foundation for selecting anticancer treatments and managing AEs in real-world practice. Chemotherapy and targeted therapy are still mainstream treatments in colorectal cancer, one of the most common malignancies worldwide. Safety is one of the leading factors in clinical decision-making, affecting patient quality of life and the benefit-risk ratio.

This article retrospectively analyzed 160 publications that met the inclusion criteria and showed that the RP of SAEs in phase III colorectal cancer clinical trials was only 25.5%, significantly lower than that of AEs, which was reported to be 96% in cancer clinical trials in a retrospective study (Sivendran et al., 2014). One of the reasons for the low RP of SAEs was insufficient attention to SAEs reports. Some researchers believed that systematic and complete SAEs reporting increased the workload and costs when the purpose of a clinical trial was only to verify drug efficacy (Wallace et al., 2016). Therefore, inadequate research funding was the other reason (Wallace et al., 2016).

Most publications included in this article did not report the type or prognosis of SAEs in detail. This was similar to a study examining the quality of SAEs reporting to sponsors by investigators from all clinical trials performed at Limoges University Hospital in 2012 (Crépin et al., 2016). In this study, 3.6% of the reports did not describe the seriousness of the SAEs, 9.3% were missing a causality assessment, and the date of SAEs onset was not mentioned in 5.7% of the reports. This phenomenon may be due to the lack of standard guidelines for SAEs reporting in clinical trials. On the other hand, the journal’s word count requirements may limit the author’s ability to provide a detailed SAEs description. The severity and duration of SAEs directly affect the prognosis and quality of life of patients, and both are essential factors for SAEs reports (Sartor, 2017). Detailed descriptions of the manifestation, severity, duration, and outcome of SAEs in phase III clinical trials, whose results have important reference value for clinical guidelines, have crucial guiding significance for real-world clinical practice. Therefore, in the future, journals about SAEs and SAEs case reports should be established for reporting SAEs in detail to better guide clinical practice and drug research and development, thereby improving cancer treatments and maximizing the benefits of patients.

The manifestations of AEs in patients with colorectal cancer were related to the drugs. The skin reactions reported in this article were more common for anti-EGFR antibodies, such as cetuximab and panitumumab, which was similar to previous reports. Some reviews and phase II clinical trials showed that the incidence of AEs and grade 3/4 AEs were 66.7% (Lynch et al., 2007) and 8%*-*16% (Soeda et al., 2014; Soda et al., 2015), respectively, for patients treated with cetuximab and 74.7% (Bouché et al., 2019) and 9%*-*15% (Nishi et al., 2016; Munemoto et al., 2018), respectively, for panitumumab. HFS was more common for regorafenib and capecitabine in this study. It has been reported that the incidence of HFS and grade 3/4 HFS were 65–69% and 15–16%, respectively, for regorafenib (Bekaii-Saab et al., 2019), and the incidence of grade 3/4 HFS for capecitabine was 8% (Soda et al., 2015) in non-phase III clinical trials. This study showed that regorafenib was related to hypertension and dyspnea, whose previously reported incidences were 62%*–*70% and 19%*–*23%, respectively, and the incidences above grade 3 were 7%*–*15% and 4%*–*6%, respectively (Bekaii-Saab et al., 2019). The incidences of hypertension, proteinuria, gastrointestinal perforation, and thrombosis were more common for bevacizumab, which was consistent with the results of many phase II clinical trials (Chen et al., 2006; Horita et al., 2011; Hong et al., 2012; Nakayama et al., 2012).

**FIGURE 5:**
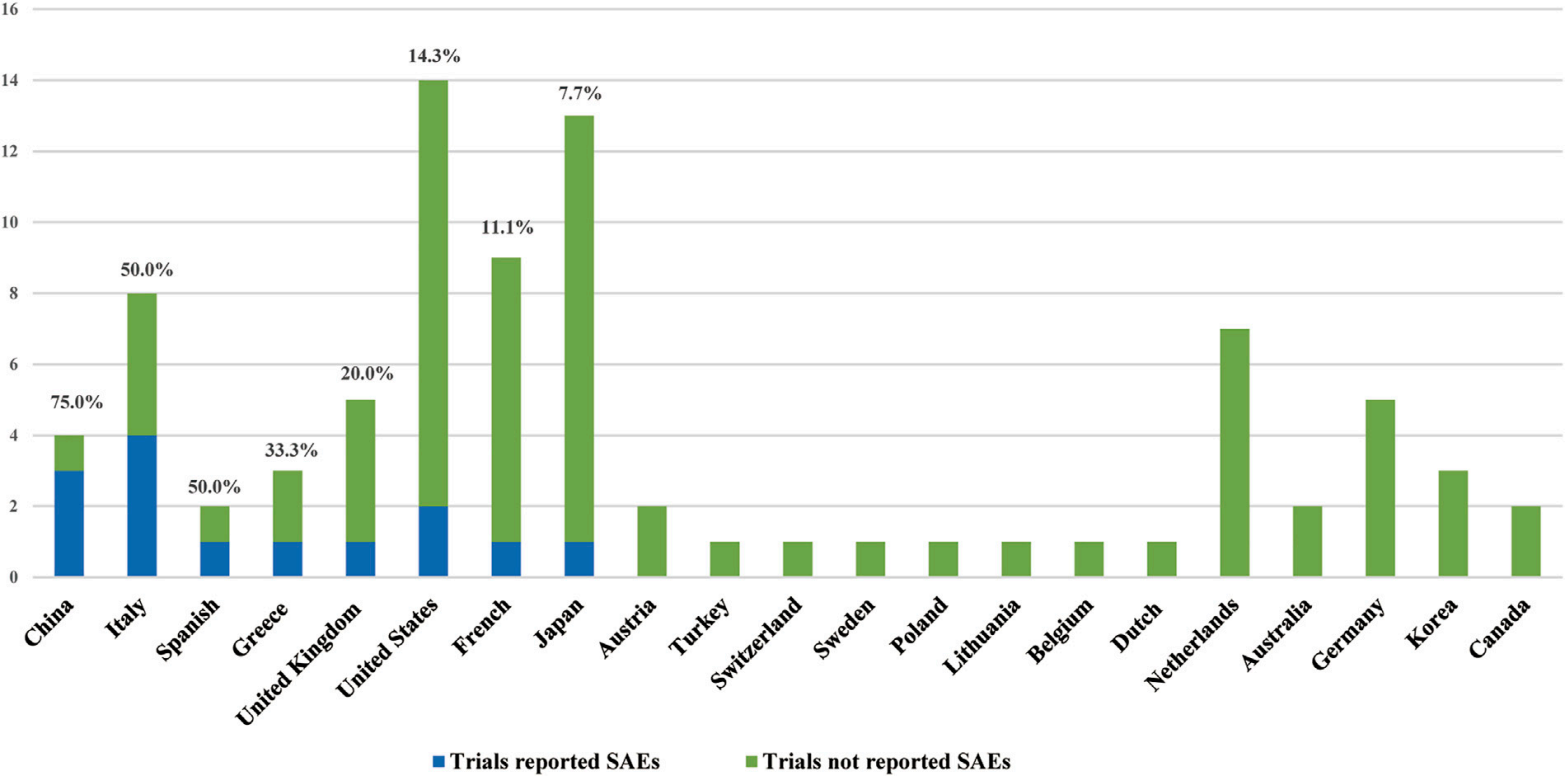
Countries and SAEs reported status.

The chi-square analysis in this study showed that the RP of SAEs in clinical trials conducted worldwide (52.4%) was higher than that in those conducted in local region (21.6%). The worldwide clinical research is supervised and reviewed by an international ethics committee and global regulatory agencies. The management system is stricter, so the reporting of SAEs is more stringent. In addition, clinical studies conducted in only one country had various reports of SAEs. The RP of SAEs was higher in China, Spain, Italy, and Greece, at 75, 50, 50, and 33.3%, respectively (Figure 5). This may be related to the differences in supervision and management of clinical research in different regions and the differences in policies and regulations.

The RP of SAEs in new drug clinical research (45.5%) was significantly higher than that in non-new drug clinical studies (15.2%) (*p* < 0.001). In addition to the effectiveness of new drugs, the safety of new drugs was of paramount concern, so the RP of SAEs was higher. Non-new drug research mainly compared the efficacy of different treatment regimens and paid less attention to SAEs, and the RP of SAEs was lower. The SAEs reporting rate of clinical studies initiated by pharmaceutical companies (57.6%) was higher than that of investigators (20.7%) (*p* < 0.001). Among 33 clinical studies initiated by pharmaceutical companies, 69.7% (23) were new drug-related clinical studies, while only 39% (32 of 82) of clinical studies undertaken by investigators were new drug studies. The RP of SAEs in new drug clinical research was higher, so the RP of SAEs in clinical research initiated by pharmaceutical companies was higher. This was also the reason why the RP of SAEs was higher in clinical trials about second line and above treatment, and higher in targeted therapy based clinical trials.

This study showed that the RP of SAEs increased in the past 26 years, which may be attributed to the following. First, the National Health and Medical Research Council has provided increasingly rigorous regulations about how SAEs should be reported (Wallace et al., 2016). Second, the increasing attention paid to drug research safety has promoted the monitoring and management of data for clinical trials (Bhattacharyya et al., 2018). Pharmaceutical companies and journal editors have made recommendations on AEs (including SAE) reporting after a thorough discussion on how policies and guidelines were followed, what challenges existed, and how challenges should be addressed to improve AEs and SAEs reporting in clinical research publications to enhance the degree of authenticity and accuracy of clinical trial data (Lineberry et al., 2016). Third, the training recommendations in the Good Clinical Practice (GCP) guidelines require investigators and study coordinators executing a clinical trial to undergo training on GCP principles every 3 years (Shanley et al., 2017), enhancing investigators’ compliance with GCP (Kuusisto et al., 2011). Finally, SAEs reports are processed by an automated computer system instead of personal reports with the development of information technology, saving workforce resources and time and facilitating the analysis of reporting performance and the nature of SAEs reports (London et al., 2009; Pecoraro and Luzi, 2011). AEs capture and management systems for cancer clinical trials were set up to administer and manage clinical trials, improving the efficiency, accuracy, and safety of AEs reports (Lencioni et al., 2015).

The top five journals for RPs of SAEs were N Engl J Med, Lancet, Lancet Oncol, JAMA, J Clin Oncol, with an average RP of SAEs 31.9%, which was significantly higher than that of other journals (16.7%, *p =* 0.030). This was affected by the journal’s requirements. For example, Lancet has provided readers with links to websites that published clinical trial protocols since 2009, and J Clin Oncol has disclosed agreements that were previously only open to journal editors and reviewers since 2011 (Song and Kim, 2020). The improvement of clinical trial transparency is beneficial to the authenticity of clinical research data.

Patients in some clinical trials completed electronic surveys regarding symptomatic AEs according to the Patient-Reported Outcomes version of the Common Terminology Criteria for Adverse Events (PRO-CTCAE) (Hagelstein et al., 2016) of the National Cancer Institute (NCI) during cancer treatment, which was demonstrated to be both feasible and informative (Chung et al., 2019). A pooled analysis showed that in oncology clinical trials, PRO and AEs reports had a different focus and were complementary (Atherton et al., 2015). Other systematic reviews showed that reported agreement between CTCAE and PRO ratings was poor to moderate in most trials (Atkinson et al., 2016). They provided evidence that PROs provided unique, valuable information that can complement CTCAE ratings, avoiding loss of AEs information because of a long interval between visits (Atkinson et al., 2016). The PRO-CTCAE included a rigorous method for capturing patient self-reports of symptomatic AEs in cancer clinical trials (Hagelstein et al., 2016) but has not been used worldwide. The effective combination of the PRO-CTCAE and clinician-reported CTCAE may be better for the management of AEs, especially SAEs, in cancer patients.

**FIGURE 6:**
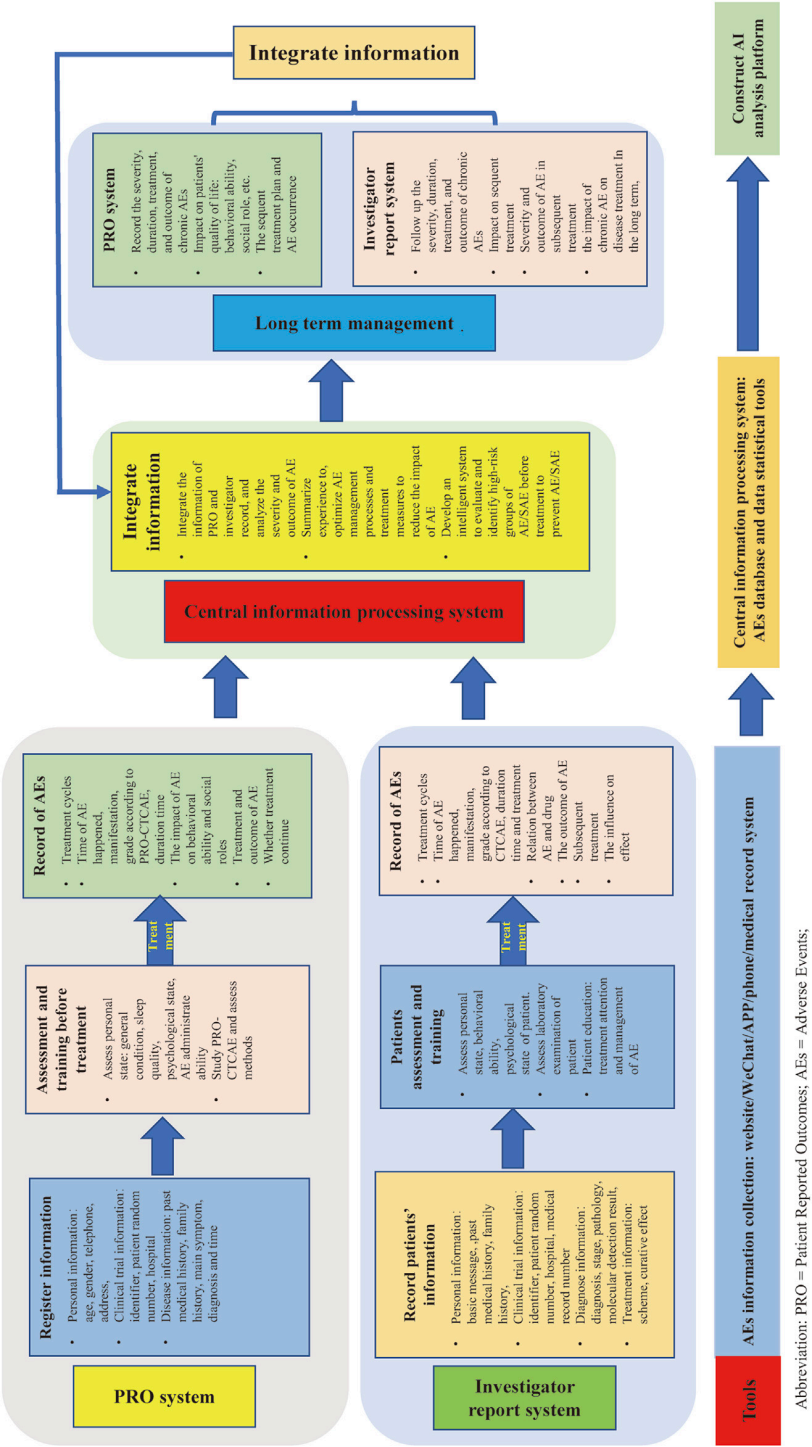
PRO and investigator system AEs comprehensive reporting process.

SAEs reports need more improvement. For example, improving the construction of SAEs reporting systems in electronic information platforms, establishing precise process and data collection methods, strengthening the training of medical staff, enhancing the safety ability assessment of patients via patient education, and improving the awareness and attention of SAEs have been reported. The authors believe that co-report of AEs/SAEs via PRO and researchers in clinical trials should be adopted in the future (Figure 6).

There were some shortcomings in this study. First, this was a retrospective study, and there may be omissions in data collection and selection bias. Second, the identification criteria for SAEs may vary because of the diverse designs of clinical trials and different judgment criteria of investigators. Third, bias existed in the data collection because the descriptions of SAEs in the publications were inconsistently attributed to the journal-specific publication requirements. Finally, this study’s included publications were all published clinical trials, and unpublished clinical trials, such as clinical trials with negative research results, were excluded. The reporting methods for SAEs have gradually improved as people pay increasing attention to SAEs. Independent reporting of SAEs by patients and researchers may better guide clinical practice and drug development in the future.

## Conclusion

In conclusion, our findings showed that the RP of SAEs increased and aroused more researchers’ attention over time. However, more efforts should be made to improve the RP of SAEs and the quality of SAEs reporting. The patterns and outcomes of SAEs should be reported in detail and given more attention to better guide drug application by clinicians in the real world. In addition, independent reporting of SAEs by patients and researchers should be encouraged.

## Data Availability

The raw data supporting the conclusion of this article will be made available by the authors, without undue reservation.
