# Supplementary material for: Serious Adverse Events Reporting in Phase III Randomized Clinical Trials of Colorectal Cancer Treatments: A Systematic Analysis
Source: Front Pharmacol. 2021 Nov 18;12:754858. doi: 10.3389/fphar.2021.754858 (PMC8636814; doi:10.3389/fphar.2021.754858)
Supplement: Supplementary file 1 [file DataSheet1.docx]

**Supplementary Material**

**References for the 160 Randomized Clinical Trials Included in the Analysis (publications No. 1-41 have reported SAEs).**

1. Peeters, M., Price, T.J., Cervantes, A., Sobrero, A.F., Ducreux, M., Hotko, Y., et al. (2010). Randomized phase III study of panitumumab with fluorouracil, leucovorin, and irinotecan (FOLFIRI) compared with FOLFIRI alone as second-line treatment in patients with metastatic colorectal cancer. *J Clin Oncol.* 28(31), 4706-4713. doi:10.1200/jco.2009.27.6055

2. Group, Q.C. (2007). Adjuvant chemotherapy versus observation in patients with colorectal cancer: a randomised study. *Lancet.* 370(9604), 2020-2029. doi:10.1016/s0140-6736(07)61866-2

3. Papadimitriou, C.A., Papakostas, P., Karina, M., Malettou, L., Dimopoulos, M.A., Pentheroudakis, G., et al. (2011). A randomized phase III trial of adjuvant chemotherapy with irinotecan, leucovorin and fluorouracil versus leucovorin and fluorouracil for stage II and III colon cancer: A Hellenic Cooperative Oncology Group study. *BMC Medicine.* 9(1). doi:10.1186/1741-7015-9-10

4. Iveson, T.J., Kerr, R.S., Saunders, M.P., Cassidy, J., Hollander, N.H., Tabernero, J., et al. (2018). 3 versus 6 months of adjuvant oxaliplatin-fluoropyrimidine combination therapy for colorectal cancer (SCOT): an international, randomised, phase 3, non-inferiority trial. *Lancet Oncol.* 19(4), 562-578. doi:10.1016/s1470-2045(18)30093-7

5. Hecht, J.R., Mitchell, E., Chidiac, T., Scroggin, C., Hagenstad, C., Spigel, D., et al. (2009). A randomized phase IIIB trial of chemotherapy, bevacizumab, and panitumumab compared with chemotherapy and bevacizumab alone for metastatic colorectal cancer.  *J Clin Oncol.* 27(5), 672-680. doi:10.1200/jco.2008.19.8135

6. Van Cutsem, E., Köhne, C.-H., Hitre, E., Zaluski, J., Chang Chien, C.-R., Makhson, A., et al. (2009). Cetuximab and chemotherapy as initial treatment for metastatic colorectal cancer. *N Engl J Med.* 360, 1408-1417. doi:10.1056/NEJMoa0805019.

7. Qin, S., Li, J., Wang, L., Xu, J., Cheng, Y., Bai, Y., et al. (2018). Efficacy and tolerability of first-line cetuximab plus leucovorin, fluorouracil, and oxaliplatin (FOLFOX-4) versus FOLFOX-4 in patients with RAS wild-type metastatic colorectal cancer: the open-label, randomized, phase III TAILOR trial. *J Clin Oncol.* 36(30), 3031-3039. doi:10.1200/jco.2018

8. Li, J., Qin, S., Xu, R.-H., Shen, L., Xu, J., Bai, Y., et al. (2018). Effect of fruquintinib vs placebo on overall survival in patients with previously treated metastatic colorectal cancer. *JAMA.* 319(24), 2486. doi:10.1001/jama.2018.7855

9. Li, J., Qin, S., Xu, R., Yau, T.C.C., Ma, B., Pan, H., et al. (2015). Regorafenib plus best supportive care versus placebo plus best supportive care in Asian patients with previously treated metastatic colorectal cancer (CONCUR): a randomised, double-blind, placebo-controlled, phase 3 trial. *Lancet Oncol.* 16(6), 619-629. doi:10.1016/s1470-2045(15)70156-7

10. Grothey, A., Van Cutsem, E., Sobrero, A., Siena, S., Falcone, A., Ychou, M., et al. (2013). Regorafenib monotherapy for previously treated metastatic colorectal cancer (CORRECT): an international, multicentre, randomised, placebo-controlled, phase 3 trial. *Lancet.* 381(26), 303-312. doi:10.1016/S0140-6736(12)61900-X

11. Douillard, J.-Y., Siena, S., Cassidy, J., Tabernero, J., Burkes, R., Barugel, M., et al. (2010). Randomized, phase III trial of panitumumab with infusional fluorouracil, leucovorin, and oxaliplatin (FOLFOX4) versus FOLFOX4 alone as first-line treatment in patients with previously untreated metastatic colorectal cancer: the PRIME study.  *J Clin Oncol.* 28(31), 4697-4705. doi:10.1200/jco.2009.27.4860

12. Hecht, J.R., Trarbach, T., Hainsworth, J.D., Major, P., Jäger, E., Wolff, R.A., et al. (2011). Randomized, placebo-controlled, phase III study of first-line oxaliplatin-based chemotherapy plus PTK787/ZK 222584, an oral vascular endothelial growth factor receptor inhibitor, in patients with metastatic colorectal adenocarcinoma.  *J Clin Oncol.* 29(15), 1997-2003. doi:10.1200/jco.2010.29.4496

13. Van Cutsem, E., Bajetta, E., Valle, J., Köhne, C.-H., Randolph Hecht, J., Moore, M., et al. (2011). Randomized, placebo-controlled, phase III study of oxaliplatin, fluorouracil, and leucovorin with or without PTK787/ZK 222584 in patients with previously treated metastatic colorectal adenocarcinoma.  *J Clin Oncol.* 29(15), 2004-2010. doi:10.1200/jco.2010.29.5436

14. Xu, J., Kim, T.W., Shen, L., Sriuranpong, V., Pan, H., Xu, R., et al. (2018). Results of a randomized, double-blind, placebo-controlled, phase III trial of trifluridine/tipiracil (TAS-102) monotherapy in Asian patients with previously treated metastatic colorectal cancer: the TERRA study. *J Clin Oncol.* 36(4), 350-358. doi:10.1200/jco.2017

15. Van Hazel, G.A., Heinemann, V., Sharma, N.K., Findlay, M.P.N., Ricke, J., Peeters, M., et al. (2016). SIRFLOX: randomized phase III trial comparing first-line mFOLFOX6 (plus or minus bevacizumab) versus mFOLFOX6 (plus or minus bevacizumab) plus selective internal radiation therapy in patients with metastatic colorectal cancer.  *J Clin Oncol.* 34(15), 1723-1731. doi:10.1200/jco.2015.66.1181

16. Cascinu, S., Rosati, G., Nasti, G., Lonardi, S., Zaniboni, A., Marchetti, P., et al. (2017). Treatment sequence with either irinotecan/cetuximab followed by FOLFOX-4 or the reverse strategy in metastatic colorectal cancer patients progressing after first-line FOLFIRI/bevacizumab: An Italian Group for the Study of Gastrointestinal Cancer phase III, randomised trial comparing two sequences of therapy in colorectal metastatic patients. *Eur J Cancer.* 83, 106e115. doi:10.1016/j.ejca.2017.06.029

17. Saini, A., Norman, A.R., Cunningham, D., Chau, I., Hill, M., Tait, D., et al. (2003). Twelve weeks of protracted venous infusion of fluorouracil (5-FU) is as effective as 6 months of bolus 5-FU and folinic acid as adjuvant treatment in colorectal cancer. *Brit J Cancer.* 88(12), 1859-1865. doi:10.1038/sj.bjc.6600995

18. Kerr, R.S., Love, S., Segelov, E., Johnstone, E.C., Falcon, B., Hewett, P., et al. (2016). Adjuvant capecitabine plus bevacizumab versus capecitabine alone in patients with colorectal cancer (QUASAR 2): an open-label, randomised phase 3 trial. *Lancet Oncol.* 17, 1543-1557. doi:10.1016/S1470-2045(16)30172-3

19. Fields, A.L.A., Keller, A., Schwartzberg, L., Bernard, S., Kardinal, C., Cohen, A., et al. (2009). Adjuvant therapy with the monoclonal antibody Edrecolomab plus fluorouracil-based therapy does not improve overall survival of patients with stage III colon cancer. *J Clin Oncol.* 27(12), 1941-1947. doi:10.1200/jco.2008.18.5710

20. Li, J., Xu, R., Qin, S., Liu, T., Pan, H., Xu, J., et al. (2018). Aflibercept plus FOLFIRI in Asian patients with pretreated metastatic colorectal cancer: a randomized Phase III study. *Future Oncol.* 14(20), 2031-2044. doi:10.2217/fon-2017-0669

21. Cunningham, D., Lang, I., Marcuello, E., Lorusso, V., Ocvirk, J., Shin, D.B., et al. (2013). Bevacizumab plus capecitabine versus capecitabine alone in elderly patients with previously untreated metastatic colorectal cancer (AVEX): an open-label, randomised phase 3 trial. *Lancet Oncol* 14, 1077-1085. doi:doi: 10.1016/S1470-2045(13)70154-2

22. De Gramont, A., Van Cutsem, E., Schmoll, H.-J., Tabernero, J., Clarke, S., Moore, M.J., et al. (2012). Bevacizumab plus oxaliplatin-based chemotherapy as adjuvant treatment for colon cancer (AVANT): a phase 3 randomised controlled trial. *Lancet Oncol.* 13(12), 1225-1233. doi:10.1016/s1470-2045(12)70509-0

23. Hoff, P.M., Hochhaus, A., Pestalozzi, B.C., Tebbutt, N.C., Li, J., Kim, T.W., et al. (2012). Cediranib plus FOLFOX/CAPOX versus placebo plus FOLFOX/CAPOX in patients with previously untreated metastatic colorectal cancer: a randomized, double-blind, phase III study (HORIZON II). *J Clin Oncol.* 30(29), 3596-3603. doi:10.1200/jco.2012.42.6031

24. Schmoll, H.-J., Cunningham, D., Sobrero, A., Karapetis, C.S., Rougier, P., Koski, S.L., et al. (2012). Cediranib with mFOLFOX6 versus bevacizumab with mFOLFOX6 as first-line treatment for patients with advanced colorectal cancer: a double-blind, randomized phase III study (HORIZON III). *J Clin Oncol.* 30, 3588-3595. doi:10.1200/JCO.2012.42.5355

25. Bennouna, J., Sastre, J., Arnold, D., sterlund, P., Greil, R., Van Cutsem, E., et al. (2013). Continuation of bevacizumab after first progression in metastatic colorectal cancer (ML18147): a randomised phase 3 trial. *Lancet Oncol.* 14, 29-37. doi:10.1016/S1470-2045(12)70477-1

26. Masi, G., Salvatore, L., Boni, L., Loupakis, F., Cremolini, C., Fornaro, L., et al. (2015). Continuation or reintroduction of bevacizumab beyond progression to first-line therapy in metastatic colorectal cancer: final results of the randomized BEBYP trial. *Ann Oncol.* 26: , 724-730. doi:10.1093/annonc/mdv012

27. Guan, Z.Z., Xu, J.M., Luo, R.C., Feng, F.Y., Wang, L.-W., Shen, L., et al. (2011). Efficacy and safety of bevacizumab plus chemotherapy in Chinese patients with metastatic colorectal cancer: a randomized phase III ARTIST trial. *Chin J Cancer.* 30(10), 682-689. doi:10.5732/cjc.011.10188

28. Sobrero, A.F., Maurel, J., Fehrenbacher, L., Scheithauer, W., Abubakr, Y.A., Lutz, M.P., et al. (2008). EPIC: phase III trial of cetuximab plus irinotecan after fluoropyrimidine and oxaliplatin failure in patients with metastatic colorectal cancer. *J Clin Oncol.* 26(14), 2311-2319. doi:10.1200/jco.2007.13.1193

29. Wasan, H.S., Gibbs, P., Sharma, N.K., Taieb, J., Heinemann, V., Ricke, J., et al. (2017). First-line selective internal radiotherapy plus chemotherapy versus chemotherapy alone in patients with liver metastases from colorectal cancer (FOXFIRE, SIRFLOX, and FOXFIRE-Global): a combined analysis of three multicentre, randomised, phase 3 trials. *Lancet Oncol* 18, 1159-1171. doi:10.1016/S1470-2045(17)30457-6

30. Eduardo, D.-R., Auxiliadora, G.-E., Bartomeu, M., Javier, S., Albert, A., Manuel, V., et al. (2012). First-line XELOX plus bevacizumab followed by XELOX plus bevacizumab or single-agent bevacizumab as maintenance therapy in patients with metastatic colorectal cancer: the phase III MACRO TTD study. *Oncologist.* 17, 15-25. doi:10.1634/theoncologist.2011-0249

31. Tournigand, C., André, T., Achille, E., Lledo, G., Flesh, M., Mery-Mignard, D., et al. (2004). FOLFIRI followed by FOLFOX6 or the reverse sequence in advanced colorectal cancer: a randomized GERCOR study. *J Clin Oncol.* 22(2), 229-237. doi:10.1200/jco.2004.05.113

32. Loupakis, F., Cremolini, C., Masi, G., Lonardi, S., Zagonel, V., Salvatore, L., et al. (2014). Initial therapy with FOLFOXIRI and bevacizumab for metastatic colorectal cancer. *N Engl J Med.* 371(23), 1609-1618. doi:10.1056/NEJMoa1403108

33. Xu, R.-H., Muro, K., Morita, S., Iwasa, S., Han, S.W., Wang, W., et al. (2018). Modified XELIRI (capecitabine plus irinotecan) versus FOLFIRI (leucovorin, fluorouracil, and irinotecan), both either with or without bevacizumab, as second-line therapy for metastatic colorectal cancer (AXEPT): a multicentre, open-label, randomised, non-inferiority, phase 3 trial. *Lancet Oncol.* 19(5), 660-671. doi:10.1016/s1470-2045(18)30140-2

34. Van Cutsem, E., Yoshino, T., Lenz, H.J., Lonardi, S., Falcone, A., Limo´N, M.L., et al. (2018). Nintedanib for the treatment of patients with refactory metastatic colorectal cancer (LUME-Colon 1): a phase III, international, randomized, placebo-controlled study. *Ann Oncol.* 29, 1955–1963. doi:10.1093/annonc/mdy241

35. Porschen, R., Arkenau, H.-T., Kubicka, S., Greil, R., Seufferlein, T., Freier, W., et al. (2007). Phase III study of capecitabine plus oxaliplatin compared with fluorouracil and leucovorin plus oxaliplatin in metastatic colorectal cancer: a final report of the AIO Colorectal Study Group. *J Clin Oncol.* 25(27), 4217-4223. doi:10.1200/jco.2006.09.2684

36. Köhne, C.H., Van Cutsem, E., Wils, J., Bokemeyer, C., El-Serafi, M., Lutz, M.P., et al. (2005). Phase III study of weekly high-dose infusional fluorouracil plus folinic acid with or without irinotecan in patients with metastatic colorectal cancer: European Organisation for Research and Treatment of Cancer Gastrointestinal Group Study 40986. *J Clin Oncol.* 23(22), 4856-4865. doi:10.1200/jco.2005.05.546

37. Lonardi, S., Sobrero, A., Rosati, G., Di Bartolomeo, M., Ronzoni, M., Aprile, G., et al. (2016). Phase III trial comparing 3–6 months of adjuvant FOLFOX4/XELOX in stage II–III colon cancer: safety and compliance in the TOSCA trial. *Ann Oncol.* 27(11), 2074-2081. doi:10.1093/annonc/mdw404

38. Schmoll, H.-J., Cartwright, T., Tabernero, J., Nowacki, M.P., Figer, A., Maroun, J., et al. (2007). Phase III trial of capecitabine plus oxaliplatin as adjuvant therapy for stage III colon cancer: a planned safety analysis in 1,864 patients. *J Clin Oncol.* 26(1), 102-109. doi:10.1200/jco.2006.08.1075

39. Popov, I., Carrato, A., Sobrero, A., Vincent, M., Kerr, D., Labianca, R., et al. (2008). Raltitrexed (Tomudex®) versus standard leucovorin-modulated bolus 5-fluorouracil: Results from the randomised phase III Pan-European Trial in Adjuvant Colon Cancer 01 (PETACC-1). *Eur J Cancer.* 44(15), 2204-2211. doi:10.1016/j.ejca.2008.07.002

40. Köhne, C.-H., Bedenne, L., Carrato, A., Bouché, O., Popov, I., Gaspà, L., et al. (2013). A randomised phase III intergroup trial comparing high-dose infusional 5-fluorouracil with or without folinic acid with standard bolus 5-fluorouracil/folinic acid in the adjuvant treatment of stage III colon cancer: The Pan-European Trial in Adjuvant Colon Cancer 2 study. *Eur J Cancer.* 49(8), 1868-1875. doi:10.1016/j.ejca.2013.01.030

41. Ychou, M., Hohenberger, W., Thezenas, S., Navarro, M., Maurel, J., Bokemeyer, C., et al. (2009). A randomized phase III study comparing adjuvant 5-fluorouracil/folinic acid with FOLFIRI in patientsfoll owing complete resection of liver metastases from colorectal cancer. *Ann Oncol.* 20, 1964–1970. doi:10.1093/annonc/mdp236

42. Masi, G., Vasile, E., Loupakis, F., Cupini, S., Fornaro, L., Baldi, G., et al. (2010). Randomized trial of two induction chemotherapy regimens in metastatic colorectal cancer: an updated analysis. *J Natl Cancer Inst.* 103(1), 21-30. doi:10.1093/jnci/djq456

43. Yamada, Y., Denda, T., Gamoh, M., Iwanaga, I., Yuki, S., Shimodaira, H., et al. (2018). S-1 and irinotecan plus bevacizumab versus mFOLFOX6 or CapeOX plus bevacizumab as first-line treatment in patients with metastatic colorectal cancer (TRICOLORE): a randomized, open-label, phase III, noninferiority trial. *Ann Oncol.* 29(3), 624-631. doi:10.1093/annonc/mdx816

44. Yoshida, M., Ishiguro, M., Ikejiri, K., Mochizuki, I., Nakamoto, Y., Kinugasa, Y., et al. (2014). S-1 as adjuvant chemotherapy for stage III colon cancer: a randomized phase III study (ACTS-CC trial). *Ann Oncol.* 25(9), 1743-1749. doi:10.1093/annonc/mdu232

45. Hong, Y.S., Park, Y.S., Lim, H.Y., Lee, J., Kim, T.W., Kim, K.-P., et al. (2012). S-1 plus oxaliplatin versus capecitabine plus oxaliplatin for first-line treatment of patients with metastatic colorectal cancer: a randomised, non-inferiority phase 3 trial. *Lancet Oncol.* 13(11), 1125-1132. doi:10.1016/s1470-2045(12)70363-7

46. Zhang, R.-X., Lin, J.-Z., Lei, J., Chen, G., Li, L.-R., Lu, Z.-H., et al. (2017). Safety of intraoperative chemotherapy with 5-FU for colorectal cancer patients receiving curative resection: a randomized, , prospective, phase III IOCCRC trial (IOCCRC). *J Cancer Res Clin Oncol.* 143(12), 2581-2593. doi:10.1007/s00432-017-2489-0

47. Koopman, M., Antonini, N.F., Douma, J., Wals, J., Honkoop, A.H., Erdkamp, F.L.G., et al. (2007). Sequential versus combination chemotherapy with capecitabine, irinotecan, and oxaliplatin in advanced colorectal cancer (CAIRO): a phase III randomised controlled trial. *Lancet.* 370(9582), 135-142. doi:10.1016/s0140-6736(07)61086-1

48. Rothenberg, M.L., Oza, A.M., Bigelow, R.H., Berlin, J.D., Marshall, J.L., Ramanathan, R.K., et al. (2003). Superiority of oxaliplatin and fluorouracil-leucovorin compared with either therapy alone in patients with progressive colorectal cancer after irinotecan and fluorouracil-leucovorin: interim results of a phase III trial. *J Clin Oncol.* 21(11), 2059-2069. doi:10.1200/jco.2003.11.126

49. Dufour, P., Husseini, F., Dreyfus, B., Cure, H., Martin, C., Prevost, G., et al. (1996). 5-Fluorouracil versus 5-fluorouracil plus α-interferon as treatment of metastatic colorectal carcinoma. A randomized study. *Ann Oncol.* 7(6), 575-579. doi:10.1093/oxfordjournals.annonc.a010673

50. Saltz, L.B., Clarke, S., Díaz-Rubio, E., Scheithauer, W., Figer, A., Wong, R., et al. (2008). Bevacizumab in combination with oxaliplatin-based chemotherapy as first-line therapy in metastatic colorectal cancer: a randomized phase III study.  *J Clin Oncol.* 26(12), 2013-2019. doi:10.1200/jco.2007.14.9930

51. Mayer, R.J., Van Cutsem, E., Falcone, A., Yoshino, T., Garcia-Carbonero, R., Mizunuma, N., et al. (2015). Randomized trial of TAS-102 for refractory metastatic colorectal cancer. *N Engl J Med.* 372(20), 1909-1919. doi:10.1056/NEJMoa1414325

52. Aparicio, T., Ghiringhelli, F., Boige, V.E., Le Malicot, K., Taieb, J., Bouch´ E, O., et al. (2018). Bevacizumab maintenance versus no maintenance during chemotherapy-free Intervals in metastatic colorectal cancer: A randomized phase III trial (PRODIGE 9). *J Clin Oncol* 36, 674-681. doi:10.1200/jco.2017

53. Nitti, D., Wils, J., Sahmoud, T., Curran, D., Couvreur, M.L., Lise, M., et al. (1997). Final results of a phase III clinical trial on adjuvant intraportal infusion with heparin and 5-fluorouracil (5-FU) in resectable colon cancer (EORTC GITCCG 1983-1987). European Organization for Research and Treatment of Cancer. Gastrointestinal Tract Cancer Cooperative Group. *EurJ Cancer.* 33(8), 1209-1215. doi:10.1016/s0959-8049(97)00052-x

54. Hausmaninger, H., Moser, R., Samonigg, H., Mlineritsch, B., Schmidt, H., Pecherstorfer, M., et al. (1999). Biochemical modulation of 5-fluorouracil by leucovorin with or without interferon-alpha-2c in patients with advanced colorectal cancer: final results of a randomised phase III study. *Eur J Cancer.* 35(3), 380-385. doi:10.1016/s0959-8049(98)00397-9

55. Lorenz, M., Moller, H.-H., Schramm, H., Gassel, H.-J., Rau, H.-G., Ridwelski, K., et al. (1998). Randomized trial of surgery versus surgery followed by adjuvant hepatic arterial infusion with 5-fluorouracil and folinic acid for liver metastases of colorectal cancer. German Cooperative on Liver Metastases (Arbeitsgruppe Lebermetastasen). *Ann Surg.* 228(6), 756-762. doi:10.1097/00000658-199812000-00006

56. Van Cutsem, E., Tabernero, J., Lakomy, R., Prenen, H., Prausová, J., Macarulla, T., et al. (2012). Addition of aflibercept to fluorouracil, leucovorin, and irinotecan improves survival in a phase III randomized trial in patients with metastatic colorectal cancer previously treated with an oxaliplatin-based regimen.  *J Clin Oncol.* 30(28), 3499-3506. doi:10.1200/jco.2012.42.8201

57. Breugom, A.J., Van Gijn, W., Muller, E.W., Berglund, , Van Den Broek, C.B.M., Fokstuen, T., et al. (2015). Adjuvant chemotherapy for rectal cancer patients treated with preoperative (chemo)radiotherapy and total mesorectal excision: a Dutch Colorectal Cancer Group (DCCG) randomized phase III trial. *Ann Oncol.* 26(4), 696-701. doi:10.1093/annonc/mdu560

58. Ohwada, S., Ikeya, T., Yokomori, T., Kusaba, T., Roppongi, T., Takahashi, T., et al. (2004). Adjuvant immunochemotherapy with oral Tegafur/Uracil plus PSK in patients with stage II or III colorectal cancer: a randomised controlled study. *Brit J Cancer.* 90(5), 1003-1010. doi:10.1038/sj.bjc.6601619

59. Nordlinger, B., Rougier, P., Arnaud, J.-P., Debois, M., Wils, J., Ollier, J.-C., et al. (2005). Adjuvant regional chemotherapy and systemic chemotherapy versus systemic chemotherapy alone in patients with stage II–III colorectal cancer: a multicentre randomised controlled phase III trial. *Lancet Oncol.* 6(7), 459-468. doi:10.1016/s1470-2045(05)70222-9

60. Yalcin, S., Uslu, R., Dane, F., Yilmaz, U., Zengin, N., Buyukunal, E., et al. (2013). Bevacizumab + capecitabine as maintenance therapy after initial bevacizumab + XELOX treatment in previously untreated patients with metastatic colorectal cancer: phase III 'Stop and Go' study results--a Turkish Oncology Group Trial. *Oncology.* 85(6), 328-335. doi:10.1159/000355914

61. Koeberle, D., Betticher, D.C., Von Moos, R., Dietrich, D., Brauchli, P., Baertschi, D., et al. (2015). Bevacizumab continuation versus no continuation after first-line chemotherapy plus bevacizumab in patients with metastatic colorectal cancer: a randomized phase III non-inferiority trial (SAKK 41/06). *Ann Oncol.* 26(4), 709-714. doi:10.1093/annonc/mdv011

62. Giantonio, B.J., Catalano, P.J., Meropol, N.J., O'dwyer, P.J., Mitchell, E.P., Alberts, S.R., et al. (2007). Bevacizumab in combination with oxaliplatin, fluorouracil, and leucovorin (FOLFOX4) for previously treated metastatic colorectal cancer: results from the Eastern Cooperative Oncology Group Study E3200. *J Clin Oncol.* 25(12), 1539-1544. doi:10.1200/jco.2006.09.6305

63. Tournigand, C., Chibaudel, B., Samson, B., Scheithauer, W., Vernerey, D., Mésange, P., et al. (2015). Bevacizumab with or without erlotinib as maintenance therapy in patients with metastatic colorectal cancer (GERCOR DREAM; OPTIMOX3): a randomised, open-label, phase 3 trial. *Lancet Oncol.* 16(15), 1493-1505. doi:10.1016/s1470-2045(15)00216-8

64. Wils, J., Blijham, G.H., Wagener, T., De Greve, J., Jansen, R.L.H., Kok, T.C., et al. (2003). High-dose 5-fluorouracil plus low dose methotrexate plus or minus low-dose PALA in advanced colorectal cancer: a randomised phase II-III trial of the EORTC Gastrointestinal Group. *Eur J Cancer.* 346-352. doi:10.1016/s0959-8049(02)00662-7

65. Tol, J., Koopman, M., Cats, A., Rodenburg, C.J., Creemers, G.J.M., Schrama, J.G., et al. (2009). Chemotherapy, bevacizumab, and cetuximab in metastatic colorectal cancer. *N Engl J Med.* 360, 563-572. doi:10.1056/NEJMoa0808268

66. Ducreux, M., Malka, D., Mendiboure, J., Etienne, P.-L., Texereau, P., Auby, D., et al. (2011). Sequential versus combination chemotherapy for the treatment of advanced colorectal cancer (FFCD 2000-05): an open-label, randomised, phase 3 trial. *Lancet Oncol.* 12, 1032-1044. doi:10.1016/S1470-2045(11)70199-1

67. Bosset, J.-F., Gilles Calais, L.C., Mineur, L., Maingon, P., Radosevic-Jelic, L., Daban, A., et al. (2006). Chemotherapy with preoperative radiotherapy in rectal cancer. *N Engl J Med.* 355, 1114-1123. doi:10.1056/NEJMoa060829

68. Sebag-Montefiore, D., Stephens, R.J., Steele, R., Monson, J., Grieve, R., Khanna, S., et al. (2009). Preoperative radiotherapy versus selective postoperative chemoradiotherapy in patients with rectal cancer (MRC CR07 and NCIC-CTG C016): a multicentre, randomised trial. *Lancet.* 373, 811-820. doi:10.1016/S0140-6736(09)60484-0

69. André, T., Boni, C., Mounedji-Boudiaf, L., Navarro, M., Tabernero, J., Hickish, T., et al. (2004). Oxaliplatin, fluorouracil, and leucovorin as adjuvant treatment for colon cancer. *N Engl J Med.* 350(23), 2343-2351. doi:10.1056/NEJMoa032709

70. Kim, J.S., Kim, J.S., Cho, M.J., Yoon, W.H. and Song, K.S. (2006). Comparison of the efficacy of oral capecitabine versus bolus 5-FU in preoperative radiotherapy of locally advanced rectal cancer. *J Korean Med Sci.* 21, 52-57. doi:10.3346/jkms.2006.21.1.52

71. Adams, R.A., Meade, A.M., Seymour, M.T., Wilson, R.H., Madi, A., Fisher, D., et al. (2011). Intermittent versus continuous oxaliplatin and fluoropyrimidine combination chemotherapy for first-line treatment of advanced colorectal cancer: results of the randomised phase 3 MRC COIN trial. *Lancet Oncol.* 12(7), 642-653. doi:10.1016/S1470-2045(11)70102-4

72. Seymour, M.T., Thompson, L.C., Wasan, H.S., Middleton, G., Brewster, A.E., Shepherd, S.F., et al. (2011). Chemotherapy options in elderly and frail patients with metastatic colorectal cancer (MRC FOCUS2): an open-label, randomised factorial trial. *Lancet.* 377, 1749-1759. doi:10.1016/S0140-6736(11)60399-1

73. Pectasides, D., Papaxoinis, G., Kalogeras, K.T., Eleftheraki, A.G., Xanthakis, I., Makatsoris, T., et al. (2012). XELIRI-bevacizumab versus FOLFIRI-bevacizumab as first-line treatment in patients with metastatic colorectal cancer: a Hellenic Cooperative Oncology Group phase III trial with collateral biomarker analysis. *BMC Cancer.* 12, 271. doi:10.1186/1471-2407-12-271

74. Hurwitz, H.I., Fehrenbacher, L., Novotny, W.F., Cartwright, T., Hainsworth, J.D., Heim, W., et al. (2004). Bevacizumab plus irinotecan, fluorouracil, and leucovorin for metastatic colorectal cancer. *N Engl J Med.* 350, 2335-2342. doi:10.1056/NEJMoa032691

75. Muro, K., Boku, N., Shimada, Y., Tsuji, A., Sameshima, S., Baba, H., et al. (2010). Irinotecan plus S-1 (IRIS) versus fluorouracil and folinic acid plus irinotecan (FOLFIRI) as second-line chemotherapy for metastatic colorectal cancer: a randomised phase 2/3 non-inferiority study (FIRIS study). *Lancet Oncol.* 11, 853-860. doi:10.1016/S1470-2045(10)70181-9

76. Hamblin', T.J., Sadullahl, S., Williamson', P., Stevenson', J., Oskam, R., Palmer, P., et al. (1993). A phase-III study of recombinant interleukin 2 and 5-fluorouracil chemotherapy in patients with metastatic colorectal cancer. *Br. J. Cancer.* 68, 1186-1189. doi:10.1038/bjc.1993.501

77. Hansen, R.M., Ryan, L., Anderson, T., Krzywda, B., Quebbeman, E., Benson, A., et al. (1996). Phase III study of bolus versus infusion fluorouracil with or without cisplatin in advanced colorectal cancer. *Journal of the National Cancer Institute.* 88(10), May 15,. doi:10.1093/jnci/88.10.668

78. Labianca, R., Cascinu, S., Frontini, L., Barni, S., Fiorentini, G., Cornelia, G., et al. (1997). High- versus low-dose levo-leucovorin as a modulator of 5-fluorouracil in advanced colorectal cancer: A'GISCAD' phase III study. *Ann Oncol.* 8, 169-174. doi:10.1023/a:1008200713533

79. Fiorentini, G., Aliberti, C., Tilli, M., Mulazzani, L., Graziano, F., Giordani, P., et al. (2012). Intra-arterial infusion of irinotecan-loaded drug-eluting beads (DEBIRI) versus intravenous therapy (FOLFIRI) for hepatic metastases from colorectal cancer: final results of a phase III study. *Anticancer Res.* 32, 1387-1396.

80. Sauer, R., Becker, H., Hohenberger, W., Rödel, C., Wittekind, C., Fietkau, R., et al. (2004). Preoperative versus postoperative chemoradiotherapy for rectal cancer. *N Engl J Med.* 351(17), 1731-1740. doi:10.1056/NEJMoa040694

81. Chibaudel, B., Maindrault-Goebel, F., Lledo, G., Mineur, L., André, T., Bennamoun, M., et al. (2009). Can chemotherapy be discontinued in unresectable metastatic colorectal cancer? The GERCOR OPTIMOX2 Study. *J Clin Oncol.* 27(34), 5727-5733. doi:10.1200/jco.2009.23.4344

82. O'connell, M.J., Colangelo, L.H., Beart, R.W., Petrelli, N.J., Allegra, C.J., Sharif, S., et al. (2014). Capecitabine and oxaliplatin in the preoperative multimodality treatment of rectal cancer: surgical end points from National Surgical Adjuvant Breast and Bowel Project trial R-04.  *J Clin Oncol.* 32(18), 1927-1934. doi:10.1200/jco.2013.53.7753

83. Rothenberg, M.L., Cox, J.V., Butts, C., Navarro, M., Bang, Y.J., Goel, R., et al. (2008). Capecitabine plus oxaliplatin (XELOX) versus 5-fluorouracil/folinic acid plus oxaliplatin (FOLFOX-4) as second-line therapy in metastatic colorectal cancer: a randomized phase III noninferiority study. *Ann Oncol.* 19(10), 1720-1726. doi:10.1093/annonc/mdn370

84. Hamaguchi, T., Shimada, Y., Mizusawa, J., Kinugasa, Y., Kanemitsu, Y., Ohue, M., et al. (2018). Capecitabine versus S-1 as adjuvant chemotherapy for patients with stage III colorectal cancer (JCOG0910): an open-label, non-inferiority, randomised, phase 3, multicentre trial. *Lancet Gastroenterol.* 3(1), 47-56. doi:10.1016/s2468-1253(17)30297-2

85. Tebbutt, N.C., Wilson, K., Gebski, V.J., Cummins, M.M., Zannino, D., Van Hazel, G.A., et al. (2010). Capecitabine, bevacizumab, and mitomycin in first-line treatment of metastatic colorectal cancer: results of the Australasian Gastrointestinal Trials Group Randomized Phase III MAX Study.  *J Clin Oncol.* 28(19), 3191-3198. doi:10.1200/jco.2009.27.7723

86. Glynne-Jones, R., Counsell, N., Quirke, P., Mortensen, N., Maraveyas, A., Meadows, H.M., et al. (2014). Chronicle: results of a randomised phase III trial in locally advanced rectal cancer after neoadjuvant chemoradiation randomising postoperative adjuvant capecitabine plus oxaliplatin (XELOX) versus control. *Ann Oncol.* 25(7), 1356-1362. doi:10.1093/annonc/mdu147

87. Hurwitz, H.I., Yi, J., Ince, W., Novotny, W.F. and Rosen, O. (2009). The clinical benefit of bevacizumab in metastatic colorectal cancer is independent of K-ras mutation status: analysis of a phase III study of bevacizumab with chemotherapy in previously untreated metastatic colorectal cancer. *Oncologist.* 14(1), 22-28. doi:10.1634/theoncologist.2008-0213

88. Asmis, T.R., Powell, E., Karapetis, C.S., Jonker, D.J., Tu, D., Jeffery, M., et al. (2011). Comorbidity, age and overall survival in cetuximab-treated patients with advanced colorectal cancer (ACRC)—results from NCIC CTG CO.17: a phase III trial of cetuximab versus best supportive care. *Ann Oncol.* 22(1), 118-126. doi:10.1093/annonc/mdq309

89. Gérard, J.-P., Azria, D., Gourgou-Bourgade, S., Martel-Laffay, I., Hennequin, C., Etienne, P.-L., et al. (2010). Comparison of two neoadjuvant chemoradiotherapy regimens for locally advanced rectal cancer: results of the phase III trial ACCORD 12/0405-Prodige 2. *J Clin Oncol.* 28(10), 1638-1644. doi:10.1200/jco.2009.25.8376

90. Blanke, C.D., Shultz, J., Cox, J., Modiano, M., Isaacs, R., Kasimis, B., et al. (2002). A double-blind placebo-controlled randomized phase III trial of 5-fluorouracil and leucovorin, plus or minus trimetrexate, in previously untreated patients with advanced colorectal cancer. *Ann Oncol.* 13(1), 87-91. doi:10.1093/annonc/mdf043

91. Venook, A.P., Niedzwiecki, D., Lenz, H.-J., Innocenti, F., Fruth, B., Meyerhardt, J.A., et al. (2017). Effect of first-line chemotherapy combined with cetuximab or bevacizumab on overall survival in patients with KRAS wild-type advanced or metastatic colorectal cancer. *JAMA.* 317(23), 2392. doi:10.1001/jama.2017.7105

92. Carrato, A., Swieboda-Sadlej, A., Staszewska-Skurczynska, M., Lim, R., Roman, L., Shparyk, Y., et al. (2013). Fluorouracil, leucovorin, and irinotecan plus either sunitinib or placebo in metastatic colorectal cancer: a randomized, phase III trial. *J Clin Oncol.* 31(10), 1341-1347. doi:10.1200/jco.2012.45.1930

93. Iwamoto, S., Takahashi, T., Tamagawa, H., Nakamura, M., Munemoto, Y., Kato, T., et al. (2015). FOLFIRI plus bevacizumab as second-line therapy in patients with metastatic colorectal cancer after first-line bevacizumab plus oxaliplatin-based therapy: the randomized phase III EAGLE study. *Ann Oncol.* 26(7), 1427-1433. doi:10.1093/annonc/mdv197

94. Heinemann, V., Von Weikersthal, L.F., Decker, T., Kiani, A., Vehling-Kaiser, U., Al-Batran, S.-E., et al. (2014). FOLFIRI plus cetuximab versus FOLFIRI plus bevacizumab as first-line treatment for patients with metastatic colorectal cancer (FIRE-3): a randomised, open-label, phase 3 trial. *Lancet Oncol.* 15(10), 1065-1075. doi:10.1016/s1470-2045(14)70330-4

95. Stintzing, S., Fischer Von Weikersthal, L., Decker, T., Vehling-Kaiser, U., Jäger, E., Heintges, T., et al. (2012). FOLFIRI plus cetuximab versus FOLFIRI plus bevacizumab as first-line treatment for patients with metastatic colorectal cancer–subgroup analysis of patients with KRAS: mutated tumours in the randomised German AIO study KRK-0306. *Ann Oncol.* 23(7), 1693-1699. doi:10.1093/annonc/mdr571

96. Hebbar, M., Chibaudel, B., André, T., Mineur, L., Smith, D., Louvet, C., et al. (2015). FOLFOX4 versus sequential dose-dense FOLFOX7 followed by FOLFIRI in patients with resectable metastatic colorectal cancer (MIROX): a pragmatic approach to chemotherapy timing with perioperative or postoperative chemotherapy from an open-label, randomized phase III trial. *Ann Oncol.* 26(2), 340-347. doi:10.1093/annonc/mdu539

97. Souglakos, J., Androulakis, N., Syrigos, K., Polyzos, A., Ziras, N., Athanasiadis, A., et al. (2006). FOLFOXIRI (folinic acid, 5-fluorouracil, oxaliplatin and irinotecan) vs FOLFIRI (folinic acid, 5-fluorouracil and irinotecan) as first-line treatment in metastatic colorectal cancer (MCC): a multicentre randomised phase III trial from the Hellenic Oncology Research Group (HORG). *Brit J Cancer.* 94(6), 798-805. doi:10.1038/sj.bjc.6603011

98. Kemeny, N.E., Niedzwiecki, D., Hollis, D.R., Lenz, H.-J., Warren, R.S., Naughton, M.J., et al. (2006). Hepatic arterial infusion versus systemic therapy for hepatic metastases from colorectal cancer: a randomized trial of efficacy, quality of life, and molecular markers (CALGB 9481).  *J Clin Oncol.* 24(9), 1395-1403. doi:10.1200/jco.2005.03.8166

99. Allegra, C.J., Yothers, G., O'connell, M.J., Sharif, S., Colangelo, L.H., Lopa, S.H., et al. (2009). Initial safety report of NSABP C-08: A randomized phase III study of modified FOLFOX6 with or without bevacizumab for the adjuvant treatment of patients with stage II or III colon cancer. *J Clin Oncol.* 27(20), 3385-3390. doi:10.1200/jco.2009.21.9220

100. Douillard, J.Y., Cunningham, D., Roth, A.D., Navarro, M., James, R.D., Karasek, P., et al. (2000). Irinotecan combined with fluorouracil compared with fluorouracil alone as first-line treatment for metastatic colorectal cancer: a multicentre randomised trial. *Lancet.* 355(9209), 1041-1047. doi:10.1016/s0140-6736(00)02034-1

101. Köhne, C.H., De Greve, J., Hartmann, J.T., Lang, I., Vergauwe, P., Becker, K., et al. (2008). Irinotecan combined with infusional 5-fluorouracil/folinic acid or capecitabine plus celecoxib or placebo in the first-line treatment of patients with metastatic colorectal cancer. EORTC study 40015. *Ann Oncol.* 19(5), 920-926. doi:10.1093/annonc/mdm544

102. Saltz, L.B., Niedzwiecki, D., Hollis, D., Goldberg, R.M., Hantel, A., Thomas, J.P., et al. (2007). Irinotecan fluorouracil plus leucovorin is not superior to fluorouracil plus leucovorin alone as adjuvant treatment for stage III colon cancer: results of CALGB 89803.  *J Clin Oncol.* 25(23), 3456-3461. doi:10.1200/jco.2007.11.2144

103. Yamada, Y., Takahari, D., Matsumoto, H., Baba, H., Nakamura, M., Yoshida, K., et al. (2013). Leucovorin, fluorouracil, and oxaliplatin plus bevacizumab versus S-1 and oxaliplatin plus bevacizumab in patients with metastatic colorectal cancer (SOFT): an open-label, non-inferiority, randomised phase 3 trial. *Lancet Oncol.* 14(13), 1278-1286. doi:10.1016/s1470-2045(13)70490-x

104. Bujko, K., Wyrwicz, L., Rutkowski, A., Malinowska, M., Pietrzak, L., Kryński, J., et al. (2016). Long-course oxaliplatin-based preoperative chemoradiation versus 5 × 5 Gy and consolidation chemotherapy for cT4 or fixed cT3 rectal cancer: results of a randomized phase III study. *Ann Oncol.* 27(5), 834-842. doi:10.1093/annonc/mdw062

105. Hegewisch-Becker, S., Graeven, U., Lerchenmüller, C.A., Killing, B., Depenbusch, R., Steffens, C.-C., et al. (2015). Maintenance strategies after first-line oxaliplatin plus fluoropyrimidine plus bevacizumab for patients with metastatic colorectal cancer (AIO 0207): a randomised, non-inferiority, open-label, phase 3 trial. *Lancet Oncol.* 16(13), 1355-1369. doi:10.1016/s1470-2045(15)00042-x

106. Simkens, L.H.J., Van Tinteren, H., May, A., Ten Tije, A.J., Creemers, G.-J.M., Loosveld, O.J.L., et al. (2015). Maintenance treatment with capecitabine and bevacizumab in metastatic colorectal cancer (CAIRO3): a phase 3 randomised controlled trial of the Dutch Colorectal Cancer Group. *Lancet.* 385(9980), 1843-1852. doi:10.1016/s0140-6736(14)62004-3

107. De Placido, S., Lopez, M., Carlomagno, C., Paoletti, G., Palazzo, S., Manzione, L., et al. (2005). Modulation of 5-fluorouracil as adjuvant systemic chemotherapy in colorectal cancer: the IGCS-COL multicentre, randomised, phase III study. *Brit J Cancer.* 93(8), 896-904. doi:10.1038/sj.bjc.6602800

108. Douillard, J.Y., Hoff, P.M., Skillings, J.R., Eisenberg, P., Davidson, N., Harper, P., et al. (2002). phase III study of uracil/tegafur and oral leucovorin versus fluorouracil and leucovorin in patients with previously untreated metastatic colorectal cancer. *J Clin Oncol.* 20(17), 3605-3616. doi:10.1200/jco.2002.04.123

109. Portier, G., Elias, D., Bouche, O., Rougier, P., Bosset, J.-F., Saric, J., et al. (2006). randomized trial of adjuvant fluorouracil and folinic acid compared with surgery alone after resection of colorectal liver metastases: FFCD ACHBTH AURC 9002 trial.  *J Clin Oncol.* 24(31), 4976-4982. doi:10.1200/jco.2006.06.8353

110. Van Cutsem, E., Peeters, M., Siena, S., Humblet, Y., Hendlisz, A., Neyns, B., et al. (2007). Open-label phase III trial of panitumumab plus best supportive care compared with best supportive care alone in patients with chemotherapy-refractory metastatic colorectal cancer.  *J Clin Oncol.* 25(13), 1658-1664. doi:10.1200/jco.2006.08.1620

111. Tournigand, C., Cervantes, A., Figer, A., Lledo, G., Flesch, M., Buyse, M., et al. (2006). OPTIMOX1: a randomized study of FOLFOX4 or FOLFOX7 with oxaliplatin in a stop-and-Go fashion in advanced colorectal cancer--a GERCOR study.  *J Clin Oncol.* 24(3), 394-400. doi:10.1200/jco.2005.03.0106

112. Scheithauer, W., Mckendrick, J., Begbie, S., Borner, M., Burns, W.I., Burris, H.A., et al. (2003). Oral capecitabine as an alternative to i.v. 5-fluorouracil-based adjuvant therapy for colon cancer: safety results of a randomized, phase III trial. *Ann Oncol.* 14(12), 1735-1743. doi:10.1093/annonc/mdg500

113. Lembersky, B.C., Wieand, H.S., Petrelli, N.J., O'connell, M.J., Colangelo, L.H., Smith, R.E., et al. (2006). Oral uracil and tegafur plus leucovorin compared with intravenous fluorouracil and leucovorin in stage II and III carcinoma of the colon: results from National Surgical Adjuvant Breast and Bowel Project Protocol C-06.  *J Clin Oncol.* 24(13), 2059-2064. doi:10.1200/jco.2005.04.7498

114. Kuebler, J.P., Wieand, H.S., O'connell, M.J., Smith, R.E., Colangelo, L.H., Yothers, G., et al. (2007). Oxaliplatin combined with weekly bolus fluorouracil and leucovorin as surgical adjuvant chemotherapy for stage II and III colon cancer: results from NSABP C-07.  *J Clin Oncol.* 25(16), 2198-2204. doi:10.1200/jco.2006.08.2974

115. Comella, P., Massidda, B., Filippelli, G., Palmeri, S., Natale, D., Farris, A., et al. (2005). Oxaliplatin plus high-dose folinic acid and 5-fluorouracil i.v. bolus (OXAFAFU) versus irinotecan plus high-dose folinic acid and 5-fluorouracil i.v. bolus (IRIFAFU) in patients with metastatic colorectal carcinoma: a Southern Italy Cooperative Oncology Group phase III trial. *Ann Oncol.* 16(6), 878-886. doi:10.1093/annonc/mdi185

116. Taieb, J., Tabernero, J., Mini, E., Subtil, F., Folprecht, G., Van Laethem, J.-L., et al. (2014). Oxaliplatin, fluorouracil, and leucovorin with or without cetuximab in patients with resected stage III colon cancer (PETACC-8): an open-label, randomised phase 3 trial. *Lancet Oncol.* 15(8), 862-873. doi:10.1016/s1470-2045(14)70227-x

117. Seymour, M.T., Brown, S.R., Middleton, G., Maughan, T., Richman, S., Gwyther, S., et al. (2013). Panitumumab and irinotecan versus irinotecan alone for patients with KRAS wild-type, fluorouracil-resistant advanced colorectal cancer (PICCOLO): a prospectively stratified randomised trial. *Lancet Oncol.* 14(8), 749-759. doi:10.1016/s1470-2045(13)70163-3

118. Price, T.J., Peeters, M., Kim, T.W., Li, J., Cascinu, S., Ruff, P., et al. (2014). Panitumumab versus cetuximab in patients with chemotherapy-refractory wild-type KRAS exon 2 metastatic colorectal cancer (ASPECCT): a randomised, multicentre, open-label, non-inferiority phase 3 study. *Lancet Oncol.* 15(6), 569-579. doi:10.1016/s1470-2045(14)70118-4

119. Nordlinger, B., Sorbye, H., Glimelius, B., Poston, G.J., Schlag, P.M., Rougier, P., et al. (2008). Perioperative chemotherapy with FOLFOX4 and surgery versus surgery alone for resectable liver metastases from colorectal cancer (EORTC Intergroup trial 40983): a randomised controlled trial. *Lancet.* 371(9617), 1007-1016. doi:10.1016/s0140-6736(08)60455-9

120. Kim, T.W., Elme, A., Kusic, Z., Park, J.O., Udrea, A.A., Kim, S.Y., et al. (2016). A phase 3 trial evaluating panitumumab plus best supportive care vs best supportive care in chemorefractory wild-type KRAS or RAS metastatic colorectal cancer. *Brit J Cancer.* 115(10), 1206-1214. doi:10.1038/bjc.2016.309

121. Fuchs, C.S., Moore, M.R., Harker, G., Villa, L., Rinaldi, D. and Hecht, J.R. (2003). Phase III comparison of two irinotecan dosing regimens in second-line therapy of metastatic colorectal cancer. *J Clin Oncol.* 21(5), 807-814. doi:10.1200/jco.2003.08.058

122. Rao, S., Cunningham, D., De Gramont, A., Scheithauer, W., Smakal, M., Humblet, Y., et al. (2004). Phase III double-blind placebo-controlled study of farnesyl transferase inhibitor R115777 in patients with refractory advanced colorectal cancer.  *J Clin Oncol.* 22(19), 3950-3957. doi:10.1200/jco.2004.10.037

123. Kim, G.P., Sargent, D.J., Mahoney, M.R., Rowland, K.M., Philip, P.A., Mitchell, E., et al. (2009). Phase III noninferiority trial comparing irinotecan with oxaliplatin, fluorouracil, and leucovorin in patients with advanced colorectal carcinoma previously treated with fluorouracil: N9841.  *J Clin Oncol.* 27(17), 2848-2854. doi:10.1200/jco.2008.20.4552

124. Ychou, M., Raoul, J.L., Douillard, J.Y., Gourgou-Bourgade, S., Bugat, R., Mineur, L., et al. (2009). A phase III randomised trial of LV5FU2 + irinotecan versus LV5FU2 alone in adjuvant high-risk colon cancer (FNCLCC Accord02/FFCD9802). *Ann Oncol.* 20(4), 674-680. doi:10.1093/annonc/mdn680

125. Colucci, G., Gebbia, V., Paoletti, G., Giuliani, F., Caruso, M., Gebbia, N., et al. (2005). Phase III randomized trial of FOLFIRI versus FOLFOX4 in the treatment of advanced colorectal cancer: a study of the Gruppo Oncologico Dell'Italia Meridionale. *J Clin Oncol.* 23(22), 4866-4875. doi:10.1200/jco.2005.07.113

126. Siu, L.L., Shapiro, J.D., Jonker, D.J., Karapetis, C.S., Zalcberg, J.R., Simes, J., et al. (2013). Phase III randomized, placebo-controlled study of cetuximab plus brivanib alaninate versus cetuximab plus placebo in patients with metastatic, chemotherapy-refractory, wild-type K-RAS colorectal carcinoma: the NCIC Clinical Trials Group and AGITG CO.20 Trial.  *J Clin Oncol.* 31(19), 2477-2484. doi:10.1200/jco.2012.46.0543

127. Poplin, E.A., Benedetti, J.K., Estes, N.C., Haller, D.G., Mayer, R.J., Goldberg, R.M., et al. (2005). Phase III Southwest Oncology Group 9415/Intergroup 0153 randomized trial of fluorouracil, leucovorin, and levamisole versus fluorouracil continuous infusion and levamisole for adjuvant treatment of stage III and high-risk stage II colon cancer.  *J Clin Oncol.* 23(9), 1819-1825. doi:10.1200/jco.2005.04.169

128. Martenson, J.A., Willett, C.G., Sargent, D.J., Mailliard, J.A., Donohue, J.H., Gunderson, L.L., et al. (2004). Phase III study of adjuvant chemotherapy and radiation therapy compared with chemotherapy alone in the surgical adjuvant treatment of colon cancer: results of intergroup protocol 0130.  *J Clin Oncol.* 22(16), 3277-3283. doi:10.1200/jco.2004.01.029

129. Díaz-Rubio, E., Tabernero, J., Gómez-España, A., Massutí, B., Sastre, J., Chaves, M., et al. (2007). Phase III study of capecitabine plus oxaliplatin compared with continuous-infusion fluorouracil plus oxaliplatin as first-line therapy in metastatic colorectal cancer: final report of the Spanish Cooperative Group for the Treatment of Digestive Tumors Trial. *J Clin Oncol.* 25(27), 4224-4230. doi:10.1200/jco.2006.09.8467

130. Price, T.J., Ross, P.J., Hickish, T., Tait, D., Norman, A.R., Ford, H.E.R., et al. (2004). Phase III study of mitomycin-C with protracted venous infusion or circadian-timed infusion of 5-fluorouracil in advanced colorectal carcinoma. *Clinical Colorectal Cancer.* 3(4), 235-242. doi:10.3816/CCC.2004.n.004

131. Giacchetti, S., Bjarnason, G., Garufi, C., Genet, D., Iacobelli, S., Tampellini, M., et al. (2006). Phase III trial comparing 4-day chronomodulated therapy versus 2-day conventional delivery of fluorouracil, leucovorin, and oxaliplatin as first-line chemotherapy of metastatic colorectal cancer: the European Organisation for Research and Treatment of Cancer Chronotherapy Group. *J Clin Oncol.* 24(22), 3562-3569. doi:10.1200/jco.2006.06.1440

132. Hendlisz, A., Eynde, M.V.D., Peeters, M., Maleux, G., Lambert, B., Vannoote, J., et al. (2010). Phase III trial comparing protracted intravenous fluorouracil infusion alone or with yttrium-90 resin microspheres radioembolization for liver-limited metastatic colorectal cancer refractory to standard chemotherapy. *J Clin Oncol.* 28(23), 3687-3694. doi:10.1200/jco.2010.28.5643

133. Miyake, Y., Nishimura, J., Kato, T., Ikeda, M., Tsujie, M., Hata, T., et al. (2017). Phase III trial comparing UFT + PSK to UFT + LV in stage IIB, III colorectal cancer (MCSGO-CCTG). *Surgery Today.* 48(1), 66-72. doi:10.1007/s00595-017-1555-1

134. Chong, G., Bhatnagar, A., Cunningham, D., Cosgriff, T.M., Harper, P.G., Steward, W., et al. (2006). Phase III trial of 5-fluorouracil and leucovorin plus either 3H1 anti-idiotype monoclonal antibody or placebo in patients with advanced colorectal cancer. *Ann Oncol.* 17(3), 437-442. doi:10.1093/annonc/mdj090

135. Tveit, K.M., Guren, T., Glimelius, B., Pfeiffer, P., Sorbye, H., Pyrhonen, S., et al. (2012). Phase III trial of cetuximab with continuous or intermittent fluorouracil, leucovorin, and oxaliplatin (Nordic FLOX) versus FLOX alone in first-line treatment of metastatic colorectal cancer: the NORDIC-VII study.  *J Clin Oncol.* 30(15), 1755-1762. doi:10.1200/jco.2011.38.0915

136. Saltz, L., Badarinath, S., Dakhil, S., Bienvenu, B., Harker, W.G., Birchfield, G., et al. (2012). Phase III trial of cetuximab, bevacizumab, and 5-fluorouracil/leucovorin vs. FOLFOX-bevacizumab in colorectal cancer. *Clinical Colorectal Cancer.* 11(2), 101-111. doi:10.1016/j.clcc.2011.05.006

137. Smalley, S.R., Benedetti, J.K., Williamson, S.K., Robertson, J.M., Estes, N.C., Maher, T., et al. (2006). Phase III trial of fluorouracil-based chemotherapy regimens plus radiotherapy in postoperative adjuvant rectal cancer: GI INT 0144.  *J Clin Oncol.* 24(22), 3542-3547. doi:10.1200/jco.2005.04.9544

138. Falcone, A., Ricci, S., Brunetti, I., Pfanner, E., Allegrini, G., Barbara, C., et al. (2007). Phase III trial of infusional fluorouracil, leucovorin, oxaliplatin, and irinotecan (FOLFOXIRI) compared with infusional fluorouracil, leucovorin, and irinotecan (FOLFIRI) as first-line treatment for metastatic colorectal cancer: the Gruppo Oncologico Nord Ovest. *J Clin Oncol.* 25(13), 1670-1676. doi:10.1200/jco.2006.09.0928

139. Fischer Von Weikersthal, L., Schalhorn, A., Stauch, M., Quietzsch, D., Maubach, P.A., Lambertz, H., et al. (2011). Phase III trial of irinotecan plus infusional 5-fluorouracil/folinic acid versus irinotecan plus oxaliplatin as first-line treatment of advanced colorectal cancer. *Eur J Cancer.* 47(2), 206-214. doi:10.1016/j.ejca.2010.09.022

140. Pinter, T., Klippel, Z., Cesas, A., Croitoru, A., Decaestecker, J., Gibbs, P., et al. (2017). A phase III, randomized, double-blind, placebo-controlled trial of pegfilgrastim in patients receiving first-line FOLFOX/bevacizumab or FOLFIRI/bevacizumab for locally advanced or metastatic colorectal cancer: final results of the pegfilgrastim and anti-VEGF evaluation study (PAVES). *Clinical Colorectal Cancer.* 16(2), 103-114.e103. doi:10.1016/j.clcc.2016.08.008

141. Latkauskas, T., Pauzas, H., Kairevice, L., Petrauskas, A., Saladzinskas, Z., Janciauskiene, R., et al. (2016). Preoperative conventional chemoradiotherapy versus short-course radiotherapy with delayed surgery for rectal cancer: results of a randomized controlled trial. *BMC Cancer.* 16(1). doi:10.1186/s12885-016-2959-9

142. Gérard, J.-P., Conroy, T., Bonnetain, F., Bouché, O., Chapet, O., Closon-Dejardin, M.-T., et al. (2006). Preoperative radiotherapy with or without concurrent fluorouracil and leucovorin in T3-4 rectal cancers: results of FFCD 9203. *J Clin Oncol.* 24(28), 4620-4625. doi:10.1200/jco.2006.06.7629

143. Schippinger, W., Samonigg, H., Schaberl-Moser, R., Greil, R., Thödtmann, R., Tschmelitsch, J., et al. (2007). A prospective randomised phase III trial of adjuvant chemotherapy with 5-fluorouracil and leucovorin in patients with stage II colon cancer. *Brit J Cancer.* 97(8), 1021-1027. doi:10.1038/sj.bjc.6604011

144. Hospers, G.a.P., Schaapveld, M., Nortier, J.W.R., Wils, J., Van Bochove, A., De Jong, R.S., et al. (2006). Randomised phase III study of biweekly 24-h infusion of high-dose 5FU with folinic acid and oxaliplatin versus monthly plus 5-FU/folinic acid in first-line treatment of advanced colorectal cancer. *Ann Oncol.* 17(3), 443-449. doi:10.1093/annonc/mdj104

145. Tol, J., Koopman, M., Rodenburg, C.J., Cats, A., Creemers, G.J., Schrama, J.G., et al. (2008). A randomised phase III study on capecitabine, oxaliplatin and bevacizumab with or without cetuximab in first-line advanced colorectal cancer, the CAIRO2 study of the Dutch Colorectal Cancer Group (DCCG). An interim analysis of toxicity. *Ann Oncol.* 19(4), 734-738. doi:10.1093/annonc/mdm607

146. Shimada, Y., Hamaguchi, T., Mizusawa, J., Saito, N., Kanemitsu, Y., Takiguchi, N., et al. (2014). Randomised phase III trial of adjuvant chemotherapy with oral uracil and tegafur plus leucovorin versus intravenous fluorouracil and levofolinate in patients with stage III colorectal cancer who have undergone Japanese D2/D3 lymph node dissection: Final results of JCOG0205. *Eur J Cancer.* 50(13), 2231-2240. doi:10.1016/j.ejca.2014.05.025

147. Lim, S.H., Kim, T.W., Hong, Y.S., Han, S.W., Lee, K.H., Kang, H.J., et al. (2015). A randomised, double-blind, placebo-controlled multi-centre phase III trial of XELIRI/FOLFIRI plus simvastatin for patients with metastatic colorectal cancer. *Brit J Cancer.* 113(10), 1421-1426. doi:10.1038/bjc.2015.371

148. Matsuda, C., Ishiguro, M., Teramukai, S., Kajiwara, Y., Fujii, S., Kinugasa, Y., et al. (2018). A randomised-controlled trial of 1-year adjuvant chemotherapy with oral tegafur–uracil versus surgery alone in stage II colon cancer: SACURA trial. *Eur J Cancer.* 96, 54-63. doi:10.1016/j.ejca.2018.03.009

149. Goldberg, R.M., Sargent, D.J., Morton, R.F., Fuchs, C.S., Ramanathan, R.K., Williamson, S.K., et al. (2004). A randomized controlled trial of fluorouracil plus leucovorin, irinotecan, and oxaliplatin combinations in patients with previously untreated metastatic colorectal cancer. *J Clin Oncol.* 22(1), 23-30. doi:10.1200/jco.2004.09.046

150. Pectasides, D., Karavasilis, V., Papaxoinis, G., Gourgioti, G., Makatsoris, T., Raptou, G., et al. (2015). Randomized phase III clinical trial comparing the combination of capecitabine and oxaliplatin (CAPOX) with the combination of 5-fluorouracil, leucovorin and oxaliplatin (modified FOLFOX6) as adjuvant therapy in patients with operated high-risk stage II or stage III colorectal cancer. *BMC Cancer.* 15(1). doi:10.1186/s12885-015-1406-7

151. Glimelius, B., Sørbye, H., Balteskard, L., Byström, P., Pfeiffer, P., Tveit, K., et al. (2008). A randomized phase III trial comparing irinotecan in combination with the Nordic bolus 5-FU and folinic acid schedule or the bolus/infused de Gramont schedule (Lv5FU2) in patients with metastatic colorectal cancer. *Ann Oncol.* 19(5), 909-914. doi:10.1093/annonc/mdm588

152. Brændengen, M., Tveit, K.M., Berglund, , Birkemeyer, E., Frykholm, G., Påhlman, L., et al. (2008). Randomized phase III study comparing preoperative radiotherapy with chemoradiotherapy in nonresectable rectal cancer. *J Clin Oncol.* 26(22), 3687-3694. doi:10.1200/jco.2007.15.3858

153. Yamazaki, K., Nagase, M., Tamagawa, H., Ueda, S., Tamura, T., Murata, K., et al. (2016). Randomized phase III study of bevacizumab plus FOLFIRI and bevacizumab plus mFOLFOX6 as first-line treatment for patients with metastatic colorectal cancer (WJOG4407G). *Ann Oncol.* 27(8), 1539-1546. doi:10.1093/annonc/mdw206

154. Cassidy, J., Clarke, S., Díaz-Rubio, E., Scheithauer, W., Figer, A., Wong, R., et al. (2008). Randomized phase III study of capecitabine plus oxaliplatin compared with fluorouracil/folinic acid plus oxaliplatin as first-line therapy for metastatic colorectal cancer. *J Clin Oncol.* 26(12), 2006-2012. doi:10.1200/jco.2007.14.9898

155. Köhne, C.H., Wils, J., Lorenz, M., Schöffski, P., Voigtmann, R., Bokemeyer, C., et al. (2003). Randomized phase III study of high-dose fluorouracil given as a weekly 24-hour infusion with or without leucovorin versus bolus fluorouracil plus leucovorin in advanced colorectal cancer: European organization of Research and Treatment of Cancer Gastrointestinal Group Study 40952. *J Clin Oncol.* 21(20), 3721-3728. doi:10.1200/jco.2003.11.122

156. Van Cutsem, E., Labianca, R., Bodoky, G., Barone, C., Aranda, E., Nordlinger, B., et al. (2009). Randomized phase III trial comparing biweekly infusional fluorouracil/leucovorin alone or with irinotecan in the adjuvant treatment of stage III colon cancer: PETACC-3. *J Clin Oncol.* 27(19), 3117-3125. doi:10.1200/jco.2008.21.6663

157. Aparicio, T., Lavau-Denes, S., Phelip, J.M., Maillard, E., Jouve, J.L., Gargot, D., et al. (2016). Randomized phase III trial in elderly patients comparing LV5FU2 with or without irinotecan for first-line treatment of metastatic colorectal cancer (FFCD 2001–02). *Ann Oncol.* 27(1), 121-127. doi:10.1093/annonc/mdv491

158. Kwakman, J.J.M., Simkens, L.H.J., Van Rooijen, J.M., Van De Wouw, A.J., Ten Tije, A.J., Creemers, G.J.M., et al. (2017). Randomized phase III trial of S-1 versus capecitabine in the first-line treatment of metastatic colorectal cancer: SALTO study by the Dutch Colorectal Cancer Group. *Ann Oncol.* 28(6), 1288-1293. doi:10.1093/annonc/mdx122

159. Johnsson, A., Hagman, H., Frödin, J.E., Berglund, , Keldsen, N., Fernebro, E., et al. (2013). A randomized phase III trial on maintenance treatment with bevacizumab alone or in combination with erlotinib after chemotherapy and bevacizumab in metastatic colorectal cancer: the Nordic ACT Trial. *Ann Oncol.* 24(9), 2335-2341. doi:10.1093/annonc/mdt236

160. Hagman, H., Frödin, J.E., Berglund, , Sundberg, J., Vestermark, L.W., Albertsson, M., et al. (2016). A randomized study of KRAS-guided maintenance therapy with bevacizumab, erlotinib or metronomic capecitabine after first-line induction treatment of metastatic colorectal cancer: the Nordic ACT2 trial. *Ann Oncol.* 27(1), 140-147. doi:10.1093/annonc/mdv490
